# Oral administration of asparagine and 3-indolepropionic acid prolongs survival time of rats with traumatic colon injury

**DOI:** 10.1186/s40779-022-00397-w

**Published:** 2022-07-06

**Authors:** Bo Cao, Rui-Yang Zhao, Hang-Hang Li, Xing-Ming Xu, Hao Cui, Huan Deng, Lin Chen, Bo Wei

**Affiliations:** 1 Department of General Surgery, the First Medical Center, Chinese PLA General Hospital, Beijing, 100853 China; 2 Medical School of Chinese PLA, Beijing, 100853 China

**Keywords:** Traumatic colon injury, Asparagine, 3-Indolepropionic acid, Intestinal homeostasis, Intestinal microbiota, Akt signaling

## Abstract

**Background:**

Traumatic colon injury (TCI) is a common disease during wartime. Prolongation of posttraumatic survival time is an effective approach to patient outcome improvement. However, there is a lack of basic research in this field. This study aimed to elucidate the mechanisms underlying TCI progression and to develop novel regimens to buy time for TCI patients on the battlefield.

**Methods:**

A total of 669 Sprague–Dawley rats were used in this study. Surgical colon incision was performed to generate the TCI rat model. The landscape of colon microbiota compositions was depicted using 16S rRNA sequencing and metabolites in the intestinal contents were detected by metabolomics profiling. The signaling transduction in the intestinal epithelium was investigated using antibody microarrays and Western blotting. The enzyme-linked immunosorbent assay was conducted to measure the levels of interleukin-6 and tumor necrosis factor-α in intestines and plasma for the detection of inflammatory responses. Diamine oxidase, D-lactate and endotoxin in plasma and protein expression of zonula occludens 1 and occludin were selected as the indicators of intestinal barrier permeability. To investigate alterations of microbiota symbiosis, the relative abundances of specific bacterial genera were detected using quantitative real-time PCR.

**Results:**

As a type of lethal injury, TCI induced acute disruption of intestinal homeostasis, characterized by inflammatory responses, intestinal barrier hyperpermeability and microbiota dysbiosis (*P* < 0.05). Significant alterations in bacterial metabolic patterns were detected with decreases in many metabolites. After a series of screenings, we found that oral administration of asparagine (Asn) and 3-indolepropionic acid (IPA) effectively prolonged posttraumatic survival time [Asn plus IPA vs. Vehicle: hazard ratio (*HR*) = 0.105, 95% CI 0.031–0.356, *P* = 0.0003] and restored intestinal homeostasis in TCI rats (*P* < 0.05). Mechanistically, this combinational strategy protected the rats against TCI through synergistic activation of Akt signaling in the intestinal epithelium (*P* < 0.05).

**Conclusions:**

Abrupt dysregulation of intestinal homeostasis plays a critical role in the progression toward TCI-induced death. Oral administration of Asn plus IPA may serve as an effective regimen to restore intestinal functions and prolong the posttraumatic survival time.

**Supplementary Information:**

The online version contains supplementary material available at 10.1186/s40779-022-00397-w.

## Background

Abdominal trauma, mainly caused by crush injuries, gunshots and stabbings, is a type of life-threatening injury with a dramatically increased incidence and mortality rate during wartime [1–3]. Traumatic colon injury (TCI) is a frequent sequel of abdominal trauma, due to the anatomic location and volume of the colon [4, 5]. Perforation repair surgery is the mainstay of treatment [6]. Nutritional support and intensive care are also important for improving patient outcomes. These modalities require safe and well-equipped medical facilities, which cannot be guaranteed on the battlefield. Meanwhile, the available evidence suggests that the survival possibility of TCI patients will sharply decline with the passage of time, a large number of whom died due to delays in evacuation to base hospitals for appropriate treatment [7, 8]. Therefore, delaying TCI progression and extending posttraumatic survival time are vital for outcome improvement. Despite advances in first-aid training and equipment, the widely applied medical approaches on the battlefield are still basic life support and local compression bandaging. Unfortunately, the actual effects are limited by a lack of adequate operation environments and cannot meet the current salvage demand. It is urgently necessary to develop novel strategies suitable for combat application to prolong the survival time of casualties with TCI.

Intestinal homeostasis plays a critical role in maintaining human health, and its dysregulation has been proven to be associated with various types of diseases [9, 10]. Intestinal homeostasis is established by functional barriers, normal distributions of microbiota and tolerant immune responses [11]. Accumulating evidence has shown that attenuation of barrier shielding exacerbates intestinal inflammation and dysbiosis [12, 13]. These pathological changes in turn further impair barrier integrity [14], triggering a vicious cycle that further disrupts homeostasis and aggravates intestinal disorders. On the basis of current research on other types of intestinal disorders, the damage to colon wall integrity caused by TCI may lead to intestinal dysfunctions and microbiota dysbiosis. Restoration of intestinal homeostasis could be a strategy for prolonging the posttraumatic survival time of TCI patients. There is, however, a lack of strong evidence to support this speculation. Investigation into the impact of TCI on intestinal homeostasis should be conducted for a better understanding of its underlying mechanisms.

Supplementation with specific metabolites holds great promise in treating intestinal disorders. Asparagine (Asn) is one type of nonessential amino acid. In 1990, it was proven to be a stimulator of colonic cell proliferation [15]. Accumulating evidence has shown that Asn can improve the integrity of the intestinal barrier, ameliorate intestinal energy deficits and reduce inflammatory responses in weanling piglets challenged with lipopolysaccharide [16–18]. 3-indolepropionic acid (IPA) is a derivative of tryptophan metabolized by intestinal microbiota. Administration of IPA alleviates intestinal microbiota dysregulation and epithelial barrier damage caused by steatohepatitis [19]. In a model of radiation enteritis, IPA replenishment significantly extends the survival time of mice by restoring intestinal homeostasis [20]. With the rapid development of chemical engineering, the production costs of Asn and IPA are low. Administration of natural chemicals via oral routes is convenient and acceptable to recipients. Therefore, if proven effective in survival time prolongation, Asn and IPA could have enormous potential value for the development of military medicine.

In this study, we aimed to establish a kind of generation methods of the TCI animal model and investigate mechanisms underlying TCI progression. Regarding the advantages of metabolite drugs, a series of screening experiments were conducted to develop novel regimens for the delay of TCI-induced death. Furthermore, the molecular mechanisms by which combinational administration of Asn plus IPA prolongs survival time of TCI rats were also identified. This research may provide basic evidence for future research on intervention strategies that can be adapted to TCI patients on the battlefield.

## Methods

### Animal experiments

A total of 669 6-week-old male Sprague–Dawley rats were purchased from Charles River (Beijing, China) and housed under specific pathogen-free conditions. The basic characteristics of the Sprague–Dawley rats are shown in Table 1. The Sprague–Dawley rats were kept in environments with 40–70% relative humidity, ambient temperature at (22 ± 2) ℃ and a 12 h/12 h light/dark period. Sterile water and standard food were provided ad libitum. After 2 weeks of acclimatization, the rats were used for the experiments. Peripheral blood was harvested from the posterior orbital venous plexus after anesthesia. Colon tissues and contents at the indicated sites were also collected. The tissues were washed with cold phosphate buffer saline (PBS) to remove the blood. Intestinal tissues and contents were stored at − 80 °C if not used immediately. The euthanasia of rats was conducted by injection of an overdose of amobarbital sodium or a continuous flow of CO_2_ into an airtight chamber until CO_2_ asphyxia was achieved. The animal experiments were approved by the Ethics Committee of Animal Center of Chinese PLA General Hospital (2020-X6-117).

**Table 1:** Basic characteristics of Sprague–Dawley rats in this study (mean ± SD)

Item	Value range
Weight (g)	210.90 ± 8.37
Length (mm)	181.00 ± 3.67
Body temperature (℃)	38.42 ± 0.34
Heart rate (count/min)	381.10 ± 7.12
Respiratory rate (count/min)	140.10 ± 9.94
Food intake [g/(100 g∙24 h)]	6.29 ± 0.86
Water intake [ml/(100 g∙24 h)]	8.27 ± 0.97

### Generation of the TCI model

To generate a model with penetrating TCI, we first intraperitoneally injected 40 mg/kg amobarbital sodium for anesthesia. The abdominal skin was shaved and disinfected. Then, sequential incisions of the skin, fascia, muscle and peritoneum were performed. The cecum served as the anatomical marker of the operations. The injury point was in the colon, 5 cm from the base of the cecum. The colon underwent a semicircular incision of 50% circumference perpendicular to its longitudinal axis. Then, the intestine was carefully restored to its anatomical position. The abdominal lumen was closed by surgical sutures. For the rats that received the sham operation, the procedure followed the identical protocol with TCI modeling exclusive of the colon incision. The blank control subjects consisted of Sprague–Dawley rats free from all surgical procedures. They only underwent the same dose of anesthesia. To ensure consistency between groups, the operation processes were performed by the same operators. All the surgical instruments underwent autoclaved sterilization and the operation was conducted on clean benches.

### Survival outcomes and sample size calculation

The experimental groups used for survival analysis are summarized as follows. Rats were divided into three groups (*n* = 15 per group): (1) naïve; (2) sham; (3) TCI for the investigation of natural survival time of TCI rats. To screen metabolites with potentials in prolonging posttraumatic survival time, rats used for TCI modeling were respectively administrated with vehicle, Asn, lactose, indoleacetic acid (IAA) or IPA (*n* = 8 per group). Next, we aimed to compare efficacies of individual metabolite treatment and combinational regimen. TCI rats with respective treatment of vehicle, Asn, IPA or Asn plus IPA were employed for survival analysis (*n* = 10 per group). Three oral inhibitors (MK-2206, wortmannin and OSU-03012) were respectively used for confirming the critical role of Akt signaling in alleviating TCI progression. TCI rats were administrated with vehicle, inhibitor, metabolite(s) or inhibitor plus metabolite(s) for survival analysis (*n* = 15 per group).

The status of the rats was ascertained every hour after TCI modeling. The initial follow-up time was based on when the colons were incised in the TCI group, when the abdominal stitches began in the sham group, and after 10 min of anesthesia in the naïve group. We mainly aimed to investigate the effects of Asn plus IPA regimen on survival prolongation of TCI rats. Due to the lack of studies in this field, we conducted a pre-test study for sample size calculation. We comprehensively analyzed the data of pre-test study and medical demand. A hazard ratio (*HR*) of 0.15 was selected for the current study. The sample size of at least 7 rats per group was calculated as necessary for 80% power and two-sided alpha of 0.05. The calculation process was performed using PASS 15.0 (NCSS Corp.).

### Administration of metabolites and target inhibitors to Sprague–Dawley rats

The vehicle was 15% Cremophor EL (MedChemExpress, NJ, USA) and 85% sterile water. The oral gavage doses of metabolites were as follows: 2 g/kg Asn [21], 4.3 g/kg lactose [22], 40 mg/kg IAA [23] or 10 mg/kg IPA [24]. The metabolites were given to the rats daily from 14 d before TCI modeling to the experimental end or rat death. The doses of inhibitors are as follows: 120 mg/kg MK-2206 [25], 1.5 mg/kg wortmannin [26] and 100 mg/kg OSU-03012 [27]. Wortmannin and OSU-03012 were orally administered daily and MK-2206 was given three times a week from 7 d before TCI modeling to experimental end or rat death.

### 16S rRNA sequencing for intestinal microbiota

To determine the bacterial compositions in the intestinal contents, naïve, sham and TCI groups were generated with 10 rats in each group. When one rat in the cohort was dying (21 h after the operation in this study), the colonic contents of all subjects were harvested. 16S rRNA sequencing was then performed. Briefly, total genomic DNA was extracted using the OMEGA Soil DNA Kit (M5635-02, Omega Biotek, GA, USA) following the manufacturer’s instruction. PCR amplification of the bacterial 16S rRNA gene V3 – V4 region was performed with the forward primer 338F (5′-ACTCCTACGGGAGGCAGCA-3′) and the reverse primer 806R (5′-GGACTACHVGGGTWTCTAAT-3′). Library construction was conducted using the TruSeq Nano DNA LT Library Prep Kit (Illumina, USA). The sequences were then filtered, denoised, and merged. Chimeras were removed using the DADA2 plugin. Nonsingleton amplicon sequence variants were aligned with Mafft and used to construct a phylogeny with FastTree 2. Sequencing was performed using a MiSeq Reagent Kit V3 and MiSeq system. Sequence data analysis was mainly performed using the QIIME2 and R packages. A total of 6 methods were employed to compare the alpha diversity between groups, including Chao1, observed species, Simpson, Shannon, Pielou and Faith’s PD evenness indices. The principal coordinate and nonmetric multidimensional scaling (MDS) analyses were conducted to analyze the beta diversity of the samples.

### Targeted metabolomics profiling of the intestinal contents

The intestinal content samples prepared for 16S rRNA sequencing were also used for the measurement of metabolite abundances. The intestinal contents were thawed in an ice bath and homogenized with zirconium oxide beads for 3 min. Metabolites were extracted with 120 μl methanol containing an internal standard. The supernatant was transferred to 96-well plates, and derivatization was carried out at 30 ℃ for 60 min. The samples were quantitated using ultra-performance liquid chromatography coupled to a tandem mass spectrometry (UPLC-MS/MS) system (ACQUITY UPLC-Xevo TQ-S, Waters Corp., Milford, MA, USA). The Metabo-Profile LIMS system was responsible for sample control and data tracking. The iMAP platform (v1.0, Metabo-Profile, Shanghai, China) was used for statistical analysis.

### Antibody microarray screening

To elucidate the mechanisms of Asn and IPA in improving intestinal homeostasis, CSP100 Plus Antibody Array (Full Moon Biosystems, Silicon Valley, USA) was used to detect protein expression and phosphorylation in the intestinal mucosa. Briefly, 9 rats were randomly divided into three groups and underwent TCI modeling. Meanwhile, the rats were treated with vehicle, Asn or IPA as described above. The colonic mucosa was collected after 24 h of TCI modeling, lysed with extraction buffer and purified using centrifugation. The lysate samples were suspended in labeling buffer, quantified by Pierce BCA Kit (Thermo Scientific, MA, USA) and labeled with biotin and Cy3-streptavidin. SureScan Microarray Scanner (Agilent, CA, USA) was used to scan the luminescent intensities of microarrays. The antibody array images were analyzed to compare the protein expression and phosphorylation.

### Quantitative real-time PCR (qRT-PCR)

qRT-PCR was performed to investigate the relative abundances of the bacterial genera. Preparation of naïve, sham and TCI models with 15 rats in each group was conducted to identify the mechanisms underlying TCI progression. The following groups were set up for identifying the effects of Asn and IPA on microbiota symbiosis (*n* = 10 per group): (1) sham + vehicle; (2) TCI + vehicle; (3) TCI + Asn; (4) TCI + IPA; (5) TCI + Asn + IPA. For the confirmation of Akt signaling functions in regulating microbiota balance, the groups were divided into the following groups (*n* = 15 per group): (1) sham + vehicle; (2) TCI + vehicle; (3) TCI + metabolite(s); (4) TCI + metabolite(s) + inhibitor. Microbiota DNA was extracted from the intestinal contents with QIAamp Fast DNA Stool Mini Kit (Qiagen, Dusseldorf, Germany). Bacterial DNA was amplified with SYBR Premix Ex Taq II (TaKaRa, Japan) and Archimed X4 system (RocGene, Beijing, China). The relative abundances of the bacterial genera were determined by the threshold cycle values. The primers used in this study are listed in Additional file 1: Table S1.

### Western blotting

To investigate expression of signaling transduction proteins in intestinal epithelium, the mucosa tissues of rats with sham operation that were administrated with vehicle and TCI rats that were administrated with vehicle, Asn, IPA or Asn plus IPA (*n* = 10 per group) after 24 h of modeling were harvested. Moreover, Western blotting was also performed to determine protein expression of intestinal mucosa from rats that were used for the confirmation of Akt signaling functions. The collected tissues were lysed with radioimmunoprecipitation assay buffer (Solarbio, Beijing, China) supplemented with a protease inhibitor cocktail (Abcam, Cambridge, UK). The tissues were sufficiently ground in a homogenizer at 4 °C, and the samples were centrifuged at 12,000 × *g* and 4 °C for 5 min to remove the precipitate. The total amount of protein was determined using Pierce BCA Kit and underwent sodium dodecyl sulfate polyacrylamide gel electrophoresis. The proteins were transferred onto polyvinylidene fluoride membranes. The membranes were blocked with 5% skim milk at 25 °C for 1 h. They were incubated with primary antibodies at 4 °C overnight and secondary antibodies at 25 °C for 1 h. The blots were imaged using Pierce ECL Plus Kit (Thermo Scientific) and Tanon 4600SF (Tanon, Shanghai, China).

### Enzyme-linked immunosorbent assay (ELISA)

Intestinal tissues and peripheral blood from aforementioned rats in the qRT-PCR experiment were harvested for detection of inflammatory responses and intestinal barrier permeability using ELISA. Tissues were ground using a homogenizer (KZ-III-F, Servicebio, Wuhan, China) and reconstituted in PBS. The samples were centrifuged at 12,000 × *g* and 4 °C for 10 min, and the supernatant was collected for ELISA. Peripheral blood was centrifuged at 2000 × *g* and 4 °C for 10 min to prepare plasma samples. ELISA kits for rat tumor necrosis factor-α (TNF-α, Abbkine, Wuhan, China), interleukin-6 (IL-6, Abbkine), diamine oxidase (DAO, Enzyme-linked Biotechnology, Shanghai, China), D-lactate (Nanjing Jiancheng, Jiangsu, China) and endotoxin (Enzyme-linked Biotechnology) were used to detect the levels of the corresponding indicators according to the manufacturer’s protocols. The absorbances were measured with a microplate reader (Biotek, VT, USA). The linear standard curves were determined using standards provided by the kits. The actual concentrations of these indicators were calculated based on standard curves.

### Immunohistochemical (IHC) staining

To detect zonula occludens 1 (ZO-1) and occludin expression in colons tissues. Colon tissues collected from the rats mentioned in the qRT-PCR experiment were fixed with 4% paraformaldehyde and embedded in paraffin. They were sectioned into slides and deparaffinized. IHC staining and score evaluation were conducted as described in our previous study [28]. The stained slides were observed under a microscope (Leica, Wetzlar, Germany).

### Statistical analysis

All data were analyzed using GraphPad Prism 8 (GraphPad Software, Inc.) and SPSS (v25.0, IBM Corp.). The normality of the data distributions was confirmed using the Shapiro–Wilk test and Q-Q plots. Continuous variables with normal distributions are presented as the mean ± standard deviation (SD). Student's *t* test was used to compare the differences in two groups; One-way analysis of variance was used to compare the differences in multiple groups and Dunn’s *t* test was conducted for the post-hoc analysis. Non-normal variables are presented as median [interquartile range (IQR)], and the Mann–Whitney *U* test or Kruskal–Wallis test was used to analyze the differences. The survival comparison was conducted using survival analyses module of GraphPad Prism 8. A *P* < 0.05 was considered statistically significant.

## Results

### TCI induces acute dysfunctions of the rat intestine

**Fig. 1:**
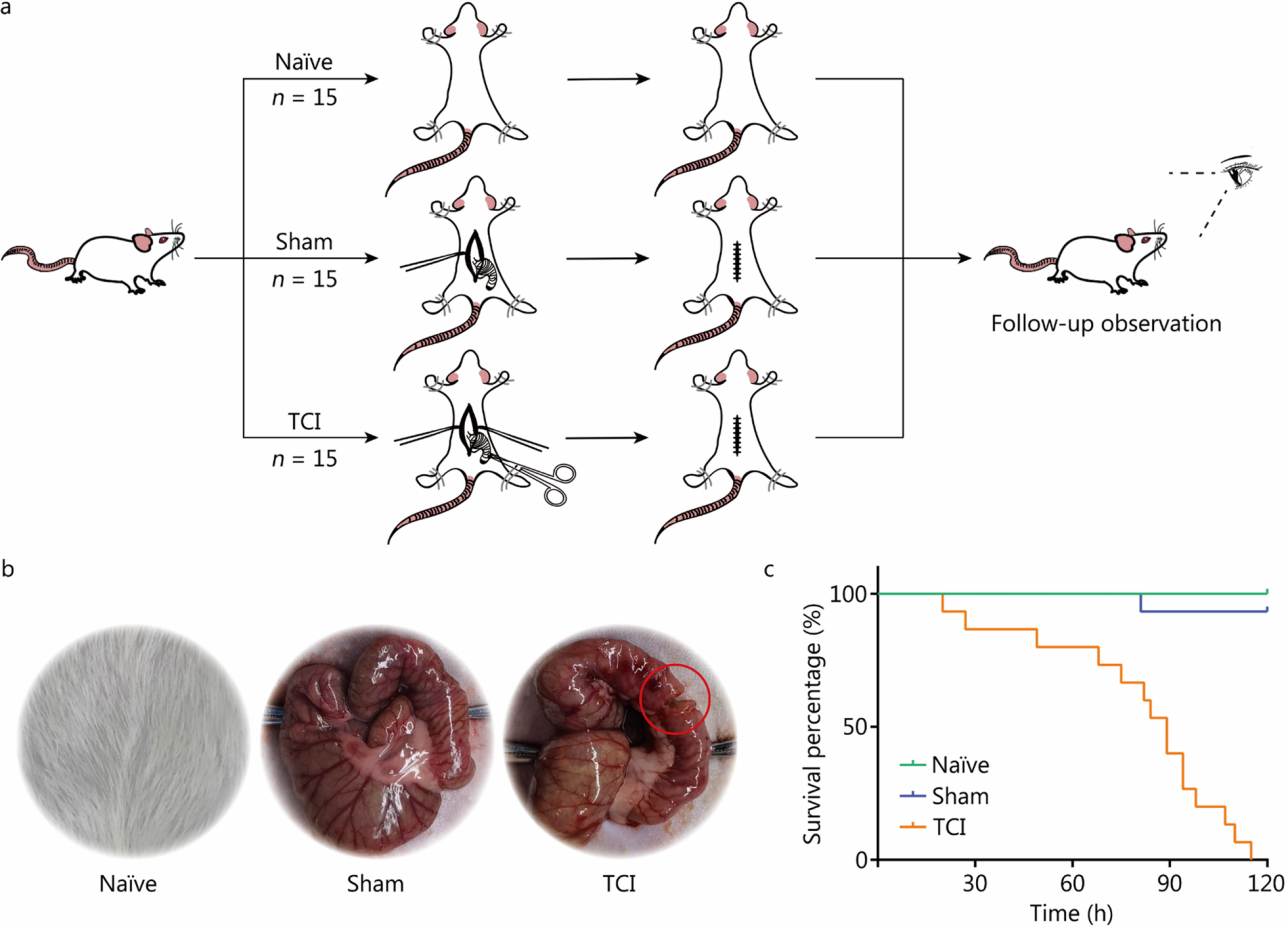
Generation of the TCI rat model. **a** The flow diagram to depict the experimental processes of preparing rats in the naïve, sham and TCI groups (*n* = 15 per group). **b** The pictures to display the operation methods of the model generation. The colon injury is marked by the red circle. **c** The survival curve to show the survival time of rats as in **a**. TCI traumatic colon injury

**Fig. 2:**
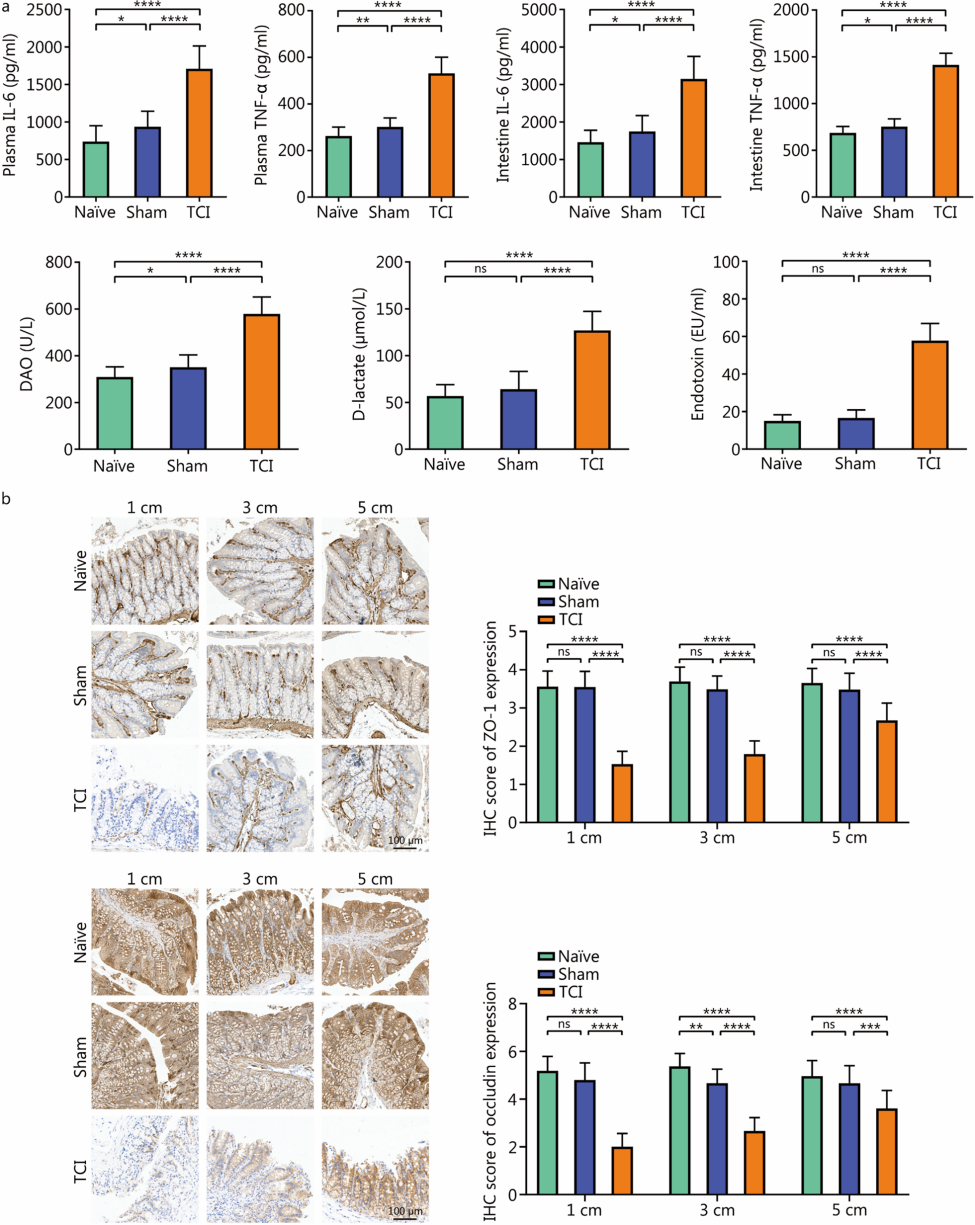
TCI induces acute dysfunctions of the rat intestine. **a** ELISA to detect indicator concentrations of rats in the naïve (*n* = 15), sham (*n* = 15) and TCI (*n* = 13) groups. **b** IHC analysis to detect ZO-1 and occludin expression in the intestinal tissues at distances of 1 cm, 3 cm and 5 cm from the lesions. The histograms of IHC scores are on the right of IHC pictures. ^*^P < 0.05, ^**^P < 0.01, ^***^P < 0.001, ^****^P < 0.0001, ns non-significant. TCI traumatic colon injury, IL-6 interleukin-6, TNF-α tumor necrosis factor-α, DAO diamine oxidase, ZO-1 zonula occludens 1, IHC immunohistochemical, ELISA enzyme-linked immunosorbent assay

To gain insights into the mechanisms underlying TCI progression, we began by creating a TCI model and investigating the survival outcomes of TCI rats (Fig. 1a, b). All TCI rats progressively died within 120 h, with a mortality rate of 100% (Fig. 1c). We next performed a series of experiments to investigate the effects of TCI on intestinal homeostasis. IL-6 and TNF-α are typical examples of multifunctional cytokines involved in inflammatory responses. Compared with the naïve and sham groups, TCI significantly increased IL-6 and TNF-α levels in both plasma and intestine tissues (*P* < 0.0001). Plasma DAO, D-lactate and endotoxin serve as indicators of intestinal permeability. Their concentrations were significantly elevated in the TCI group (*P* < 0.0001, Fig. 2a). Furthermore, IHC analysis was conducted to determine the expression of ZO-1 and occludin, which are markers of intestinal barrier permeability. ZO-1 and occludin were downregulated by TCI, and there was an appreciable distance-dependent relationship (*P* < 0.01, Fig. 2b).

### Intestinal microbiota is dramatically disrupted in TCI rats

**Fig. 3:**
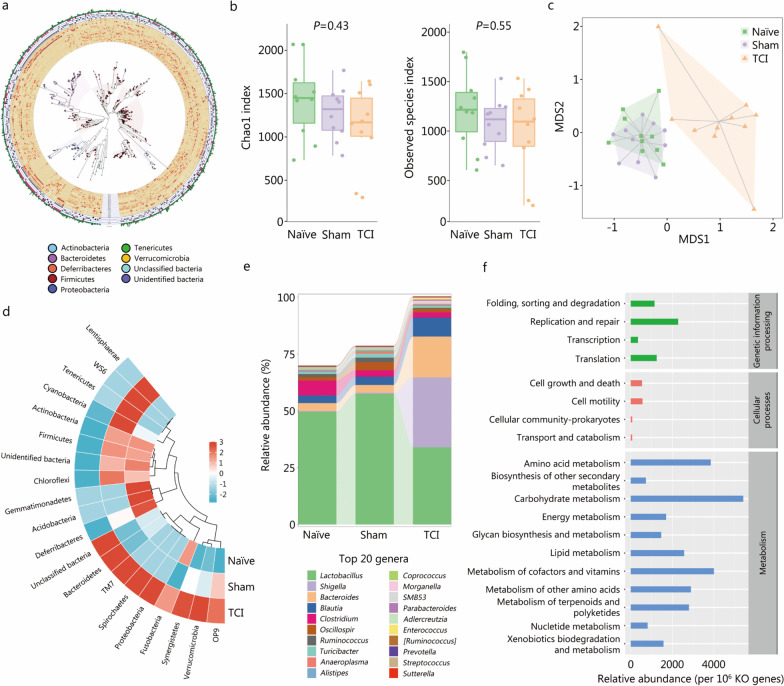
The intestinal microbiota is dramatically disrupted in TCI rats. **a** The GraPhlAn plot to display the species richness of rat intestinal microbiota in the naïve, sham and TCI groups. **b** Chao1 and observed species analysis to compare the alpha diversities. **c** The principal coordinate analysis to compare the beta diversities. **d** The heatmap to show the changes of bacterial phyla that ranked top 20 of the average abundances. **e** The stacked bar chart to display the changes of bacterial genera that ranked top 20 of the average abundances. *Ruminococcus* without square brackets refers to the bacteria that are formally classified as the *Ruminococcus* genus. *[Ruminococcus]* appertains to the bacteria that are temporarily classified as the *Ruminococcus* genus. **f** KEGG analysis to show the associated pathways in the genetic information processing, cellular processes and metabolism categories. TCI traumatic colon injury, KEGG Kyoto Encyclopedia of Genes and Genomes, MDS multidimensional scaling, KO KEGG orthology

The damage to intestinal wall integrity caused by TCI led us to wonder whether microbiota symbiosis concomitantly changes. 16S rRNA sequencing was performed to interrogate the identification profiles of the rat flora distributions. The GraPhlAn plot displayed the landscape of detectable microbiota richness at the phylum level (Fig. 3a). No significant differences in alpha diversity among the naïve, sham and TCI groups were observed (*P* > 0.05; Fig. 3b, Additional file 2: Fig. S1a-d), indicating that species evenness and the amounts were unchanged. Nevertheless, beta diversity analysis revealed that TCI rats harbored a bacterial community distinct from those in the naïve and sham groups (Fig. 3c; Additional file 2: Fig. S1e). As shown by the heatmap, TCI induced significant displacement of the dominant bacterial phyla (Fig. 3d). Specifically among the top 20 genera of the rat intestinal flora, a significant decrease in the abundances of *Lactobacillus* and *Ruminococcus* was accompanied by an increase in the abundances of *Shigella*, *Bacteroides*, *Morganella* and *Enterococcus* (*P* < 0.05, Fig. 3e). qRT-PCR analysis was performed to confirm the altered abundances of the 6 bacterial genera (*P* < 0.0001, Additional file 2: Fig. S2). Furthermore, Kyoto Encyclopedia of Genes and Genomes (KEGG) analysis was conducted to reveal the mechanistic associations of TCI-induced microbiota dysregulation. The metabolism category was predominantly enriched compared to other types of pathways, suggesting that TCI may affect the metabolic patterns of the intestinal microbiota (Fig. 3f, Additional file 2: Fig. S3).

### TCI results in significant changes in metabolic patterns of the intestinal microbiota

**Fig. 4:**
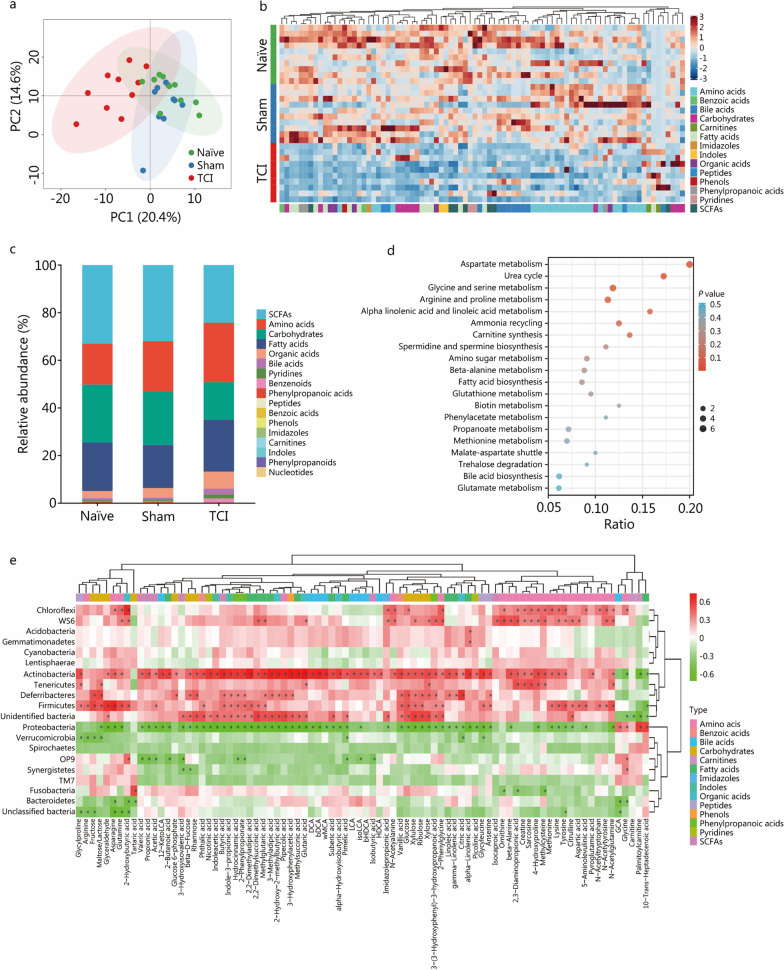
TCI results in significant changes in metabolic patterns of the intestinal microbiota. **a** The principal component analysis to display the differences of metabolite patterns of intestinal microbiota in the naïve, sham and TCI groups. **b** The heatmap to display metabolites with differential levels (*P* < 0.05). **c** The stacked bar chart to show the relative abundances of metabolite classes. **d** The bubble chart to show the enriched top 20 pathways performed by SMPDB analysis. **e** The heatmap to exhibit the correlations between bacterial distributions and differential metabolites. ^*^P < 0.05. TCI traumatic colon injury, PC principal component, SCFA short-chain fatty acid, SMPDB Small Molecule Pathway Database

Since the evidence implies that microbial metabolism may be affected, we conducted targeted metabolomics profiling to determine the relative abundances of metabolites in the colonic contents. Principal component analysis displayed a relatively smaller overlap between the TCI and sham groups than between the naïve and sham groups (Fig. 4a), suggesting a considerable impact of TCI on microbial metabolism. The heatmap displayed the wide suppression of metabolites by TCI modeling (Fig. 4b). Specifically, 5 metabolite classes accounted for the highest proportions, including short-chain fatty acids (SCFAs), amino acids, carbohydrates, fatty acids and organic acids. Concomitant with a decrease in the abundances of SCFAs and carbohydrates, the proportions of amino acids and organic acids were relatively increased (Fig. 4c). We next employed Small Molecule Pathway Database (SMPDB) analysis to illustrate the impact of TCI on metabolic patterns. As shown by the bubble chart, the top 3 terms were aspartate metabolism, urea cycle and glycine and serine metabolism. Nearly half of the metabolic pathways were enriched in amino acid metabolism (Fig. 4d). Conjoint analysis revealed close correlations between microbiota dysregulation and metabolite alterations (Fig. 4e).

### Coadministration of Asn and IPA ameliorates intestinal homeostasis dysregulation induced by TCI

The results of the microbiome and metabolomics analysis led to the speculation that supplementation with specific deficient metabolites may restore intestinal homeostasis and extend the survival time of TCI rats. We first set screening criteria to select the studied metabolites and four metabolites were eligible for further experiments, including Asn, lactose, IAA and IPA (Additional file 2: Fig. S4a). Violin plots displayed their abundances measured by metabolomics profiling (*P* < 0.05, Additional file 2: Fig. S4b). Next, TCI rats were orally administrated with the four metabolites following the protocol as shown in Additional file 2: Fig. S4c. Asn or IPA effectively prolonged the survival time of rats with TCI (Asn vs. Vehicle: *HR* = 0.171, 95% CI 0.047–0.618, *P* = 0.0071; IPA vs. Vehicle: *HR* = 0.248, 95% CI 0.072–0.855, *P* = 0.0273). However, no significant efficacy of lactose was observed (Lactose vs. Vehicle: *HR* = 0.698, 95% CI 0.243–2.004, *P* = 0.5036) and IAA treatment reduced the survival rate of the TCI rats (IAA vs. Vehicle: *HR* = 4.000; 95% CI 1.177–13.600, *P* = 0.0264) (Additional file 2: Fig. S4d).

**Fig. 5:**
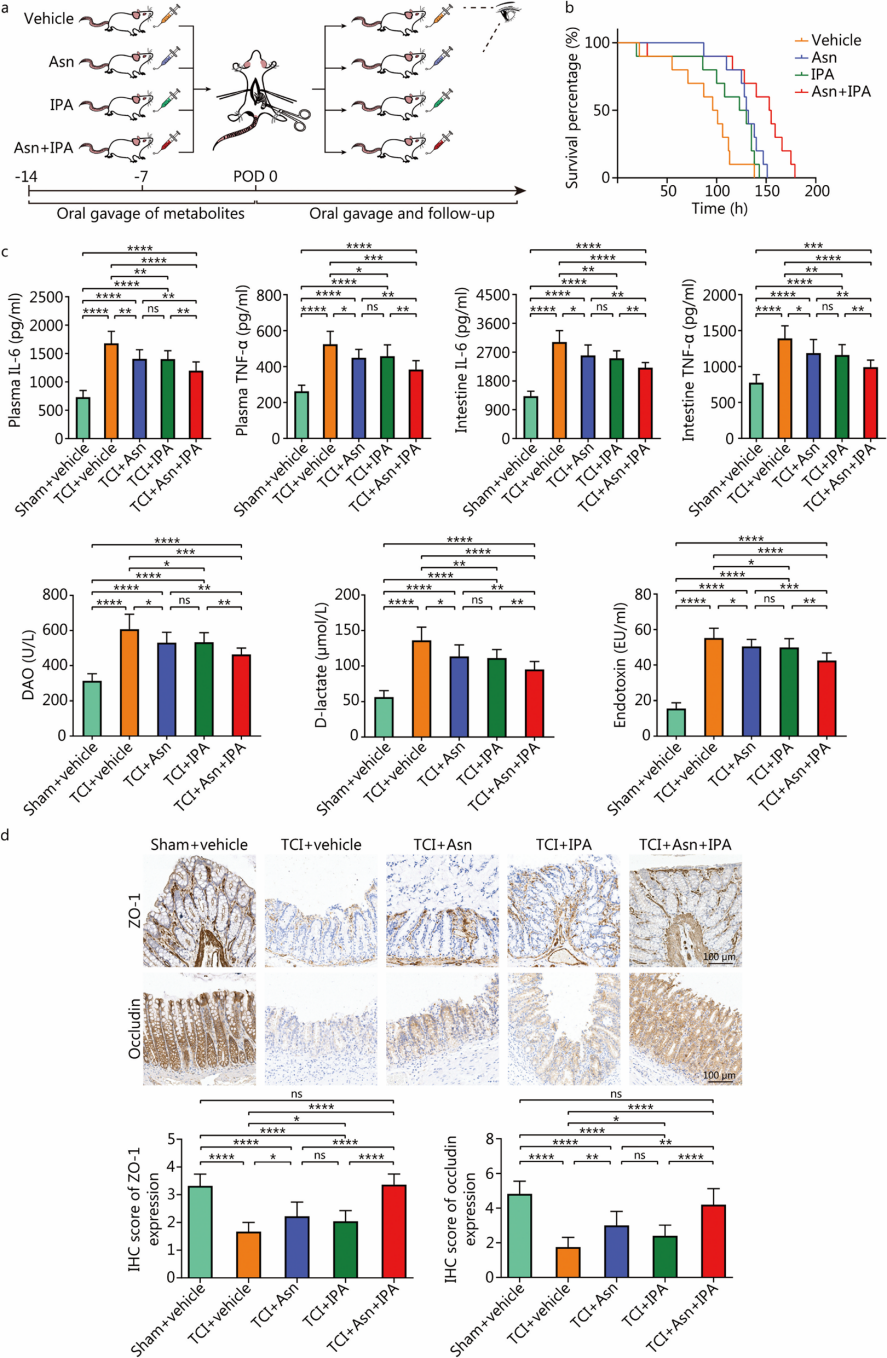
Coadministration of Asn and IPA ameliorates intestinal homeostasis dysregulation induced by TCI. **a** The flow diagram to depict the experimental processes of preparing rats with TCI that were administered with vehicle, Asn, IPA or Asn plus IPA (*n* = 10 per group). **b** The survival curve to show the survival time of the rats as in **a**. **c** ELISA to detect indicator concentrations of rats with sham operation that were administered with vehicle (*n* = 10) and TCI rats that were administered with vehicle (*n* = 9), Asn (*n* = 10), IPA (*n* = 10) or Asn plus IPA (*n* = 10). **d** The IHC analysis to detect ZO-1 and occludin expression in intestinal tissues of rats as in **c**. The histograms of IHC scores are below IHC pictures. ^*^P < 0.05, ^**^P < 0.01, ^***^P < 0.001, ^****^P < 0.0001, ns non-significant. TCI traumatic colon injury, Asn asparagine, IPA 3-indolepropionic acid, IL-6 interleukin-6, TNF-α tumor necrosis factor-α, DAO diamine oxidase, ZO-1 zonula occludens 1, ELISA enzyme-linked immunosorbent assay, IHC immunohistochemical

Given the desirable effects of Asn and IPA, we verified the efficacy of combined administration of Asn plus IPA (Fig. 5a). As expected, the Asn plus IPA regimen effectively prolonged survival time of TCI rats (Asn plus IPA vs. Vehicle: *HR* = 0.105, 95% CI 0.031–0.356, *P* = 0.0003). Furthermore, this regimen outperformed individual treatment (Asn plus IPA vs. Asn: *HR* = 0.249; 95% CI 0.082–0.755, *P* = 0.0141; Asn plus IPA vs. IPA: *HR* = 0.207, 95% CI 0.067–0.639, *P* = 0.0061) (Fig. 5b). Administration of either metabolite reduced IL-6 and TNF-α levels in both plasma and intestines, alleviated the leakage of DAO, D-lactate and endotoxin and increased the expression of ZO-1 and occludin (*P* < 0.05). Rats gavaged with Asn plus IPA experienced more restorative effects than those with individual treatment (*P* < 0.01) (Fig. 5c, d). Moreover, qRT-PCR analysis was performed to evaluate the functions of Asn plus IPA in modulating the imbalance of intestinal flora. Compared with individual treatment or control groups, relative abundances of *Lactobacillus* and *Ruminococcus* were increased after coadministration of Asn and IPA, accompanied by decreased abundances of *Bacteroides*, *Shigella*, *Morganella* and *Enterococcus* (*P* < 0.01, Additional file 2: Fig. S5).

### Asn plus IPA regimen facilitates intestinal homeostasis recovery by activating Akt signaling in the intestinal epithelium

**Fig. 6:**
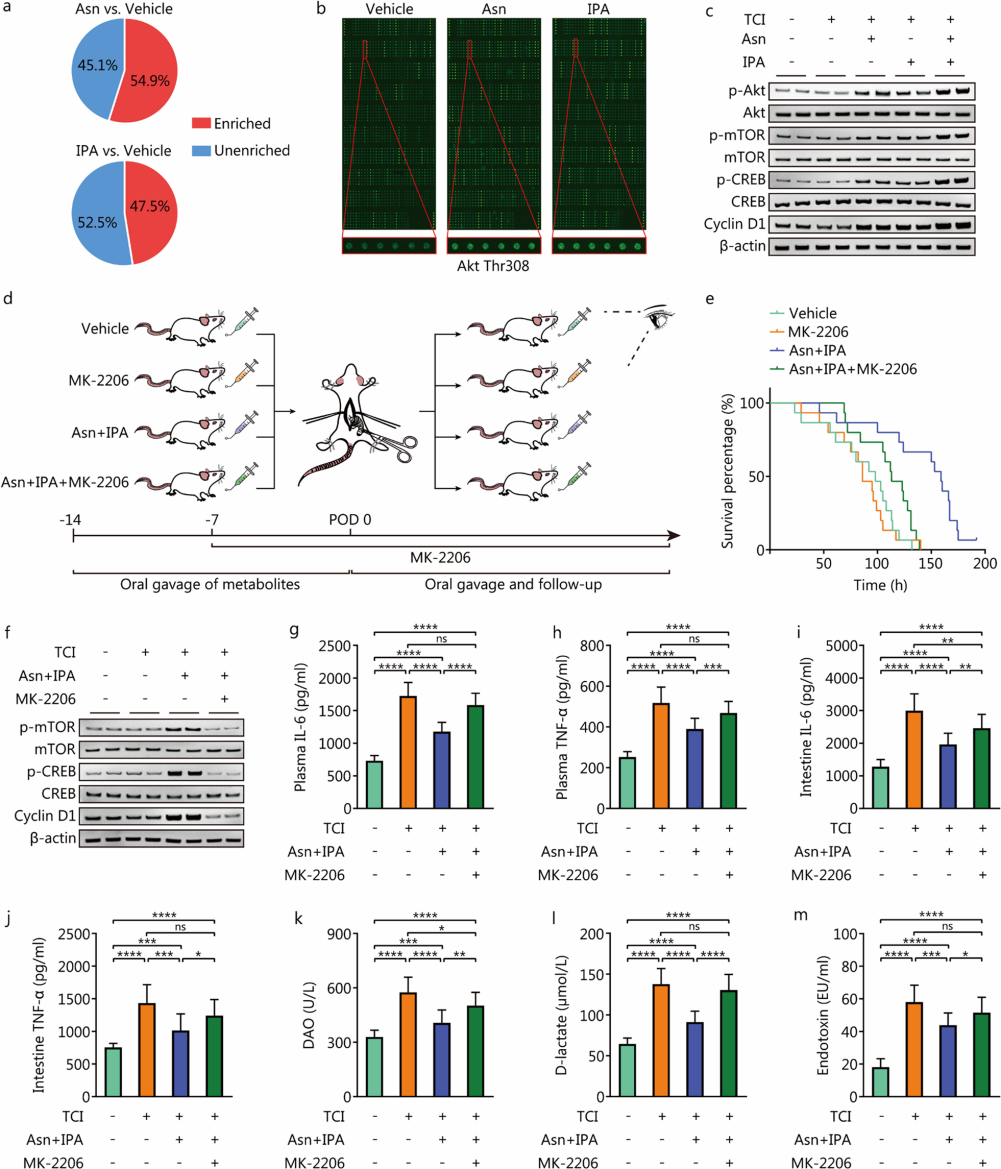
Asn plus IPA regimen facilitates intestinal homeostasis recovery by activating Akt signaling in the intestinal epithelium. **a** The pie charts to show the proportions of protein phosphorylation sites that are enriched in PI3K/Akt pathway among the sites with significantly altered phosphorylation levels (FC ≥ 1.3 or FC ≤ 0.7). **b** The antibody microarray images to show the landscape of detected protein expression and phosphorylation. Akt Thr308 phosphorylation levels are compared between vehicle, Asn and IPA groups. **c** Western blotting analysis to show protein expression and phosphorylation of intestinal epithelium of rats that received indicated treatment. **d** The flow diagram to depict the experimental processes of preparing rats with TCI that were administered with vehicle, MK-2206, Asn plus IPA or Asn plus IPA and MK-2206 (*n* = 15 per group). **e** The survival curve to show the survival time of the rats as in **d**. **f** Western blotting analysis to show protein expression and phosphorylation of intestinal epithelium of rats with sham operation that were administered with vehicle (*n* = 15) and TCI rats that were administered with vehicle (*n* = 12), Asn plus IPA (*n* = 15) or Asn plus IPA and MK-2206 (*n* = 13). **g**–**m** The ELISA to detect indicator concentrations of rats as in **f**. ^*^P < 0.05, ^**^P < 0.01, ^***^P < 0.001, ^****^P < 0.0001, ns non-significant. TCI traumatic colon injury, Asn asparagine, IPA 3-indolepropionic acid, IL-6 interleukin-6, TNF-α tumor necrosis factor-α, DAO diamine oxidase, mTOR mammalian target of rapamycin, CREB cAMP-response element binding protein, ELISA enzyme-linked immunosorbent assay, POD post-operative day

To delve more deeply into the mechanisms underlying the efficacy of Asn plus IPA treatment, we employed antibody microarrays to depict the landscapes of the alterations of key signaling pathways in the intestinal epithelium. Approximately half of the phosphorylation sites with significant changes (FC ≥ 1.3 or FC ≤ 0.7) were enriched in phosphoinositide 3-kinase (PI3K)/Akt signaling after Asn or IPA treatment (Fig. 6a). Akt, as a critical target in this pathway, was significantly phosphorylated at the Thr308 site after administration of the two metabolites (*P* < 0.05, Fig. 6b), suggesting that Asn and IPA may both serve as Akt activators in the intestinal epithelium of TCI rats. The Western blotting analysis confirmed the profiling of the antibody microarrays. Furthermore, coadministration of Asn plus IPA further activated Akt compared to individual treatment, concomitant with increased phosphorylation of mammalian target of rapamycin (mTOR) and cAMP response element binding protein (CREB) and elevated expression of cyclin D1 (Fig. 6c).

To further investigate the role of Akt signaling in homeostasis restoration and verify its regulatory relationships with Asn plus IPA treatment, MK-2206, an active allosteric Akt inhibitor, was provided to block Akt activity in the intestinal epithelium (Fig. 6d). Individual administration of MK-2206 did not affect posttraumatic survival time (MK-2206 vs. Vehicle: *HR* = 1.167, 95% CI 0.553–2.467, *P* = 0.6851), whereas it drastically impaired the survival benefit from Asn plus IPA treatment (Asn + IPA + MK-2206 vs. Asn + IPA: *HR* = 4.939; 95% CI 1.950–12.510, *P* = 0.0008, Fig. 6e). The increased phosphorylation of mTOR and CREB and elevated expression of cyclin D1 were also inhibited (Fig. 6f), which confirmed the inhibitory effects of MK-2206 administration on Akt signaling inhibition. Furthermore, MK-2206 reversed the reduction in indicators of inflammatory responses and intestinal hyperpermeability induced by Asn plus IPA (*P* < 0.05; Fig. 6g–m, Additional file 2: Fig. S6a). The partial correction of gut dysbiosis was also abated (*P* < 0.0001, Additional file 2: Fig. S6b).

### Asn and IPA activate Akt signaling by phosphorylating PI3K and 3-phosphoinositide dependent kinase 1 (PDK1) respectively

Interestingly, the upstream mechanisms underlying Akt activation by Asn and IPA may be different. PI3K, a canonical activator of Akt, was phosphorylated at the Tyr607 site after Asn treatment, suggesting that Asn may activate Akt signaling by phosphorylating PI3K in the intestinal epithelium (*P* < 0.05; Additional file 2: Fig. S7a, b). To validate this speculation, we used wortmannin, an oral PI3K inhibitor, to block PI3K activity in the intestinal epithelium and found that Asn-induced survival extension was abolished (Wortmannin vs. Vehicle: *HR* = 0.930, 95% CI 0.439–1.969, *P* = 0.8496; Asn + wortmannin vs. Asn: *HR* = 3.871, 95% CI 1.595–9.394, *P* = 0.0028; Additional file 2: Fig. S7c, d). Western blotting analysis showed that wortmannin blocked the enhancement of Akt, mTOR, CREB phosphorylation and cyclin D1 expression caused by Asn (Additional file 2: Fig. S7e), which proved that gavage with wortmannin was an effective approach to PI3K inhibition. The experiments for the detection of intestinal homeostasis were repeated to investigate the inhibitory effects of wortmannin. As expected, the alleviation of inflammatory responses, intestinal barrier hyperpermeability and microbiota dysregulation was considerably antagonized by blocking PI3K activity (*P* < 0.01; Additional file 2: Fig. S7f-m, Additional file 2: Fig. S8).

**Fig. 7:**
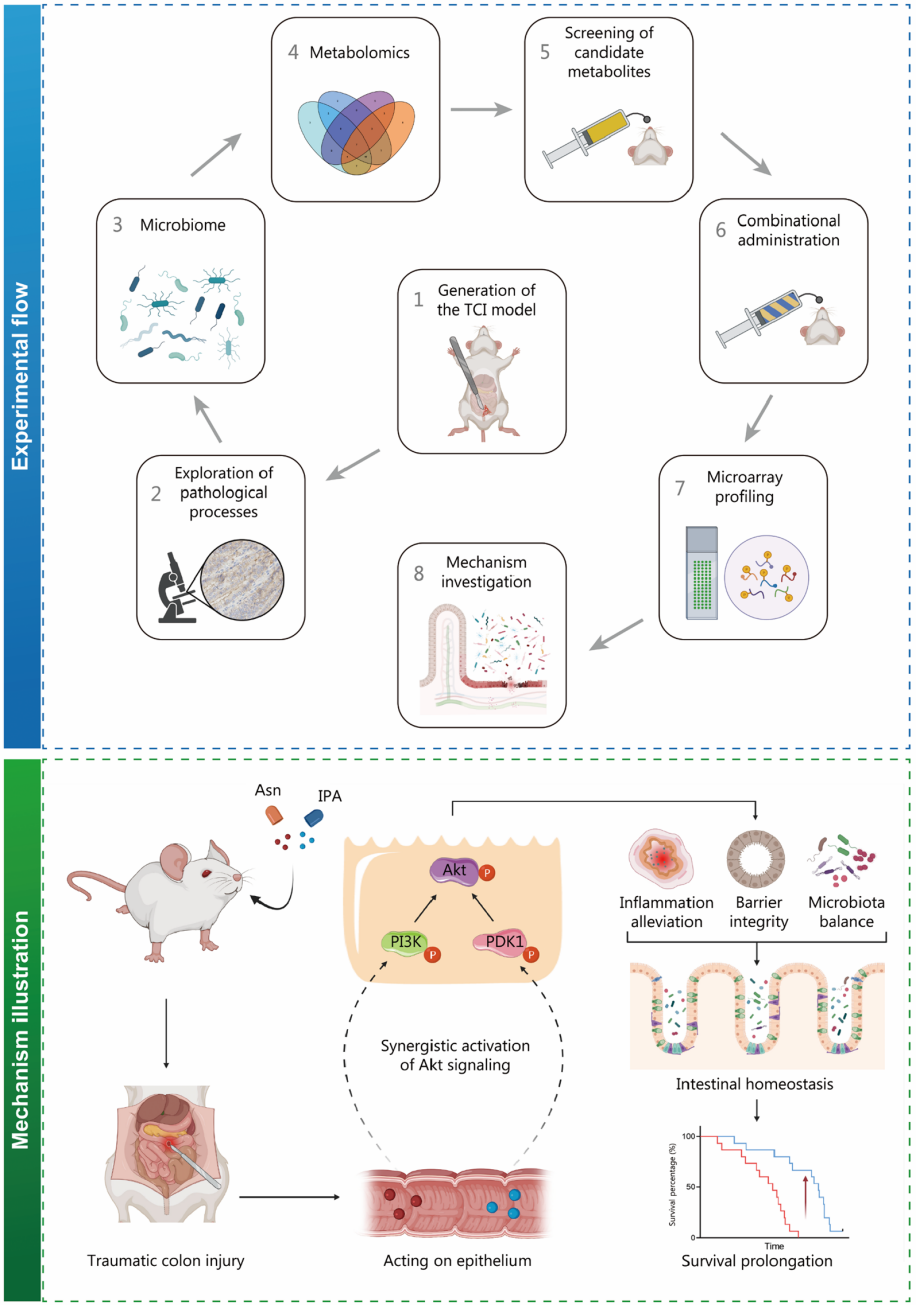
The schematic illustration of an experimental flow chart and mechanisms by which Asn plus IPA administration prolongs posttraumatic survival time of TCI rats. The whole experimental flow is displayed on the upper section (blue). The research progress is displayed by the numbers and arrow directions. The mechanisms of Asn plus IPA administration are illustrated on the lower section (green). Oral administration of Asn and IPA synergistically activates Akt signaling in intestinal epithelium. The dysregulation of intestinal homeostasis induced by TCI is alleviated, evidenced by inflammatory response alleviation, restoration of barrier integrity and microbiota rebalance. The posttraumatic survival time of TCI rats is subsequently extended. TCI traumatic colon injury, Asn asparagine, IPA 3-indolepropionic acid, PI3K phosphoinositide 3-kinase, PDK1 3-phosphoinositide dependent kinase 1

In contrast to the mechanisms of Asn, PI3K was not activated by IPA, suggesting that IPA may regulate Akt activity in a PI3K-independent manner (*P* > 0.05, Additional file 2: Fig. S9a). PDK1 is a serine/threonine protein kinase that directly phosphorylates Akt at Thr308. The antibody microarray data showed that the fold increase in PDK1 Ser241 phosphorylation ranked first among the detected sites (*P* < 0.05; Additional file 2: Fig. S9a, b). Therefore, IPA was speculated to facilitate homeostasis improvement by PDK1/Akt pathway activation. Similar experiments were performed with the administration of OSU-03012, an inhibitor of PDK1 (Additional file 2: Fig. S9c). The significant survival benefit from IPA was attenuated by OSU-03012 (OSU-03012 vs. Vehicle: *HR* = 0.944, 95% CI 0.447–1.995, *P* = 0.8808; IPA + OSU-03012 vs. IPA: *HR* = 3.430, 95% CI 1.426–8.252, *P* = 0.0059, Additional file 2: Fig. S9d). Gavage of OSU-03012 abrogated IPA-induced promotion of Akt, mTOR, CREB phosphorylation and cyclin D1 expression (Additional file 2: Fig. S9e). Amelioration of intestinal homeostasis damage caused by IPA administration was also significantly impaired by PDK1 inactivation (*P* < 0.05; Additional file 2: Fig. S9f-m, Fig. S10). In summary, our findings demonstrate that Asn and IPA synergistically enhance Akt signaling by activating PI3K and PDK1, respectively, in intestinal epithelium (Fig. 7).

## Discussion

TCI poses serious threats to warfighters due to its significantly increased incidence and mortality during wartime. Extension of the posttraumatic survival time is a key issue for increasing the survival rates of casualties with TCI. In this study, we created a rat model of penetrating TCI and found that TCI induced acute disruption of intestinal homeostasis, characterized by inflammatory responses, barrier hyperpermeability and microbiota dysbiosis. The three pathological alterations, which are important contributors to intestinal damage [29–31], collectively shortened the survival time of the TCI rats. Furthermore, TCI led to significant changes in the microbiota metabolic patterns, which prompted us to investigate the potential of these metabolites for military medical applications. We found that oral gavage of Asn plus IPA effectively restored intestinal homeostasis and extended the survival time of TCI rats in a manner highly dependent on Akt signaling. Asn and IPA respectively promoted phosphorylation of PI3K and PDK1 activity in the intestinal epithelium, giving rise to synergistic activation of Akt.

Intestinal symbiosis critically impacts human health. *Lactobacillus* is the dominant genus in the colon and it regulates the normal functions of the gut [32]. *Ruminococcus* is positively correlated with immune responses and regulates metabolic disorders [33]. Ectopic levels of *Shigella*, *Bacteroides*, *Morganella* and *Enterococcus* induce intestinal inflammation and accelerate disease progression [34–37]. The acute imbalance of beneficial and pathogenic bacteria, including these 6 genera, may serve as one of the promoters of TCI-induced death, and this dysbiosis was reversed by administration of Asn plus IPA. During the progression of intestinal disorders caused by nontraumatic factors, microbiota dysregulation also acts as an important contributor [38–40]. Restoration of microbiota symbiosis is a promising approach to disease treatment. In contrast, the bacterial functions during the progression of defined damage of the colonic mucosa are seemingly opposite, although TCI and this type of wound both pertain to mechanical intestinal injuries. The microbiota can spontaneously form definable consortia in injured sites and stimulate mucosa repair processes [41]. This contradiction may be attributable to the degrees of colon injuries. Commensal microbiota can maintain a steady state and play a positive role in injury alleviation when anatomic continuity still exists. After intestinal perforation drastically changes the microenvironment, microbiota homeostasis is disrupted and significantly promotes disease progression. This further verifies the importance of intestinal wall integrity.

The reported functions of Akt signaling in regulating intestinal homeostasis remain controversial. Our findings show that the restorative effects of Asn and IPA are mainly dependent on Akt activation. Some studies have likewise revealed the protective role of Akt signaling. For instance, ferulic acid restores heat stress-induced intestinal dysfunctions by phosphorylating Akt [42]. Resveratrol, a natural compound, also protects against oxidative stress-mediated intestinal barrier injury in a PI3K/Akt-dependent manner [43]. However, activation of PI3K/Akt signaling may contribute to the progression of dextran sodium sulfate-induced colitis [44]. This could be due to the different types of intestinal disorders and modeling methods. Furthermore, the contradiction reminds us that interventions designed to regulate signaling pathways and treat intestinal disorders may have risks in fueling other diseases. The unknown side effects of related drugs should be carefully investigated.

There are some limitations to this study. First, we invented a model with surgical incision of the colon to simulate individuals who suffer from TCI. However, TCI conditions can be affected by multiple factors, such as wound size, traumatic location, intestine displacement and harsh environment. More experiments with more gradient intervention conditions are required to investigate the pathological progression and verify the efficacies of Asn and IPA. Second, we preliminarily validated the effectiveness of Asn plus IPA gavage in prolonging the survival time of TCI rats. Optimal dosages and administration routes remain to be explored. Third, we found that block of Akt activity cannot thoroughly antagonize the efficacies of Asn plus IPA administration, suggesting unknown bypass functions in addition to the Akt signaling pathway. Further investigation should be conducted to depict the mechanistic landscape.

In summary, we created a TCI model with high repeatability and stability. Although incapable of completely simulating the TCI conditions on the battlefield, this model can provide a reference for the methodology of TCI model generation. The pathological mechanisms of TCI were elucidated by jointly using microbiome and metabolomics profiling, which can offer deep insights into pathological processes during TCI-induced death. The critical role of intestinal homeostasis in maintaining health is reaffirmed based on our findings. More importantly, administration of Asn plus IPA effectively extended the posttraumatic survival time of TCI rats, suggesting the potential value of metabolite administration in wartime. More related investigations and achievements are needed to safeguard life safety and health of military personnel.

## Conclusions

Our findings demonstrate that TCI acutely disrupts intestinal homeostasis, a major contributing factor in TCI-induced death. Oral administration of Asn and IPA ameliorates intestinal dysregulation by synergistically activating Akt signaling in the intestinal epithelium, further prolonging the survival time of rats with TCI. This medical regimen may provide a basis for further research on intervention strategies to be applied on the battlefield.

## Supplementary Information

**Additional file 1. Table S1:** Sequences of primers used in this study.

**Additional file 2. Fig. S1** Alpha and beta diversity analysis of intestinal microbiota among the naïve, sham and TCI groups. **Fig. S2** Confirmation of microbiota dysregulation induced by TCI. **Fig. S3** KEGG analysis to show the associated pathways in the environmental information processing, human diseases and organismal systems categories based on 16S rRNA sequencing data. KEGG Kyoto Encyclopedia of Genes and Genomes. **Fig. S4** Oral gavage of Asn or IPA extends the survival time of TCI rats. **Fig. S5** Administration of Asn plus IPA mitigates intestinal microbiota dysbiosis induced by TCI. **Fig. S6** Asn and IPA supplementation ameliorates intestinal hyperpermeability and intestinal microbiota dysbiosis induced by TCI through Akt activation in intestinal epithelium. **Fig. S7** Oral administration of Asn prolongs survival time and promotes intestinal recovery through activating PI3K/Akt pathway in intestinal epithelium. **Fig. S8** Asn administration mitigates intestinal microbiota dysbiosis through phosphorylating PI3K in intestinal epithelium. **Fig. S9** Oral administration of IPA prolongs survival time and restores intestinal functions through activating PDK1/Akt pathway in intestinal epithelium. **Fig. S10** IPA administration mitigates intestinal microbiota dysbiosis through phosphorylating PDK1 in intestinal epithelium.

## Data Availability

The datasets used and analyzed during the current study are available from the corresponding author on reasonable request.

## Abbreviations

Asn: Asparagine
CI: Confidence interval
CREB: CAMP response element binding protein
DAO: Diamine oxidase
ELISA: Enzyme-linked immunosorbent assay
FC: Fold change
HR: Hazard ratio
IAA: Indoleacetic acid
IHC: Immunohistochemical
IL-6: Interleukin-6
IPA: 3-Indolepropionic acid
KEGG: Kyoto Encyclopedia of Genes and Genomes
KO: KEGG orthology
MDS: Multidimensional scaling
mTOR: Mammalian target of rapamycin
PBS: Phosphate buffer saline
PDK1: 3-Phosphoinositide dependent kinase 1
PI3K: Phosphoinositide 3-kinase
POD: Post-operative day
qRT-PCR: Quantitative real-time PCR
SCFA: Short-chain fatty acid
SD: Standard deviation
SMPDB: Small Molecule Pathway Database
TCI: Traumatic colon injury
TNF-α: Tumor necrosis factor-α
ZO-1: Zonula occludens 1
