# Supplementary material for: Oral administration of asparagine and 3-indolepropionic acid prolongs survival time of rats with traumatic colon injury
Source: Mil Med Res. 2022 Jul 6;9:37. doi: 10.1186/s40779-022-00397-w (PMC9258171; doi:10.1186/s40779-022-00397-w)
Supplement: Supplementary file 1 — Additional file 1. Table S1: Sequences of primers used in this study. [file 40779_2022_397_MOESM1_ESM.pdf]

Table S1 Sequences of primers used in this study

**Bacterial genera**	**Sequence (5'-3')**
Lactobacillus	Forward: AGCAGTAGGGAATCTTCCA
Lactobacillus	Reverse: CACCGCTACACATGGAG
Shigella	Forward: CAAGTCCGTAAATTCATTCTCTCTTT
Shigella	Reverse: GCTGGAAAAACTCAGTGCCTCCC
Bacteroides	Forward: ATAGCCTTTCGAAAGTAAGAT
Bacteroides	Reverse: CCAGTATCAACTGCAATTTTA
Ruminococcus	Forward: GGTGGCAAAGCCATTCGGT
Ruminococcus	Reverse: GTTACGGGACGGTCAGAG
Morganella	Forward: GGCGGTAACAGGGAGAAGCTT
Morganella	Reverse: CGGTAACGTCAATTGCCAAGGT
Enterococcus	Forward: CCCTTATTGTTAGTTGCCATCATT
Enterococcus	Reverse: ACTCGTTGTACTTCCCATTGT
Total bacteria	Forward: AGAGTTTGATCATGGCTCAG
Total bacteria	Reverse: ACCGCGACTGCTGCTGGCAC
