# Supplementary material for: Oral administration of asparagine and 3-indolepropionic acid prolongs survival time of rats with traumatic colon injury
Source: Mil Med Res. 2022 Jul 6;9:37. doi: 10.1186/s40779-022-00397-w (PMC9258171; doi:10.1186/s40779-022-00397-w)
Supplement: Supplementary file 2 — Additional file 2. Fig. S1 Alpha and beta diversity analysis of intestinal microbiota among the naïve, sham and TCI groups. Fig. S2 Confirmation of microbiota dysregulation induced by TCI. Fig. S3 KEGG analysis to show the associated pathways in the environmental information processing, human diseases and organismal systems categories based on 16S rRNA sequencing data. KEGG Kyoto Encyclopedia of Genes and Genomes. Fig. S4 Oral gavage of Asn or IPA extends the survival time of TCI rats. Fig. S5 Administration of Asn plus IPA mitigates intestinal microbiota dysbiosis induced by TCI. Fig. S6 Asn and IPA supplementation ameliorates intestinal hyperpermeability and intestinal microbiota dysbiosis induced by TCI through Akt activation in intestinal epithelium. Fig. S7 Oral administration of Asn prolongs survival time and promotes intestinal recovery through activating PI3K/Akt pathway in intestinal epithelium. Fig. S8 Asn administration mitigates intestinal microbiota dysbiosis through phosphorylating PI3K in intestinal epithelium. Fig. S9 Oral administration of IPA prolongs survival time and restores intestinal functions through activating PDK1/Akt pathway in intestinal epithelium. Fig. S10 IPA administration mitigates intestinal microbiota dysbiosis through phosphorylating PDK1 in intestinal epithelium. [file 40779_2022_397_MOESM2_ESM.pdf]

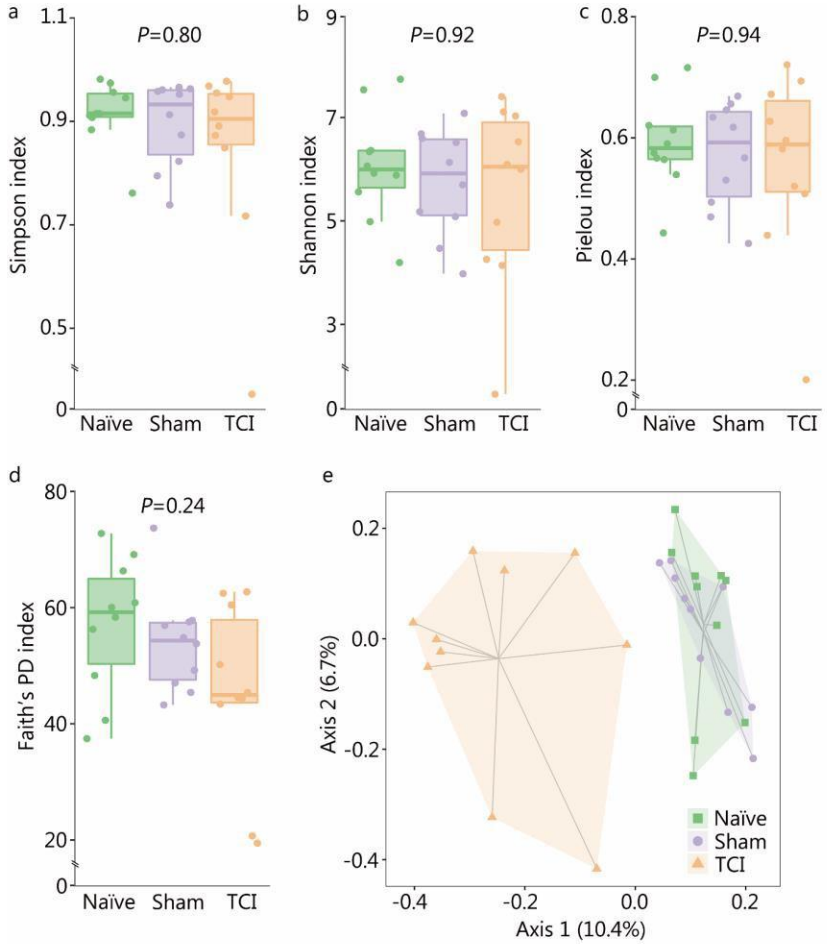

Fig. S1 Alpha and beta diversity analysis of intestinal microbiota among the naïve, sham and TCI groups. Simpson (**a**), Shannon (**b**), Pielou (**c**) and Faith's PD (**d**) analysis to compare the alpha diversities of intestinal microbiota. **e** Nonmetric multidimensional scaling to compare the beta diversities of intestinal microbiota. TCI traumatic colon injury

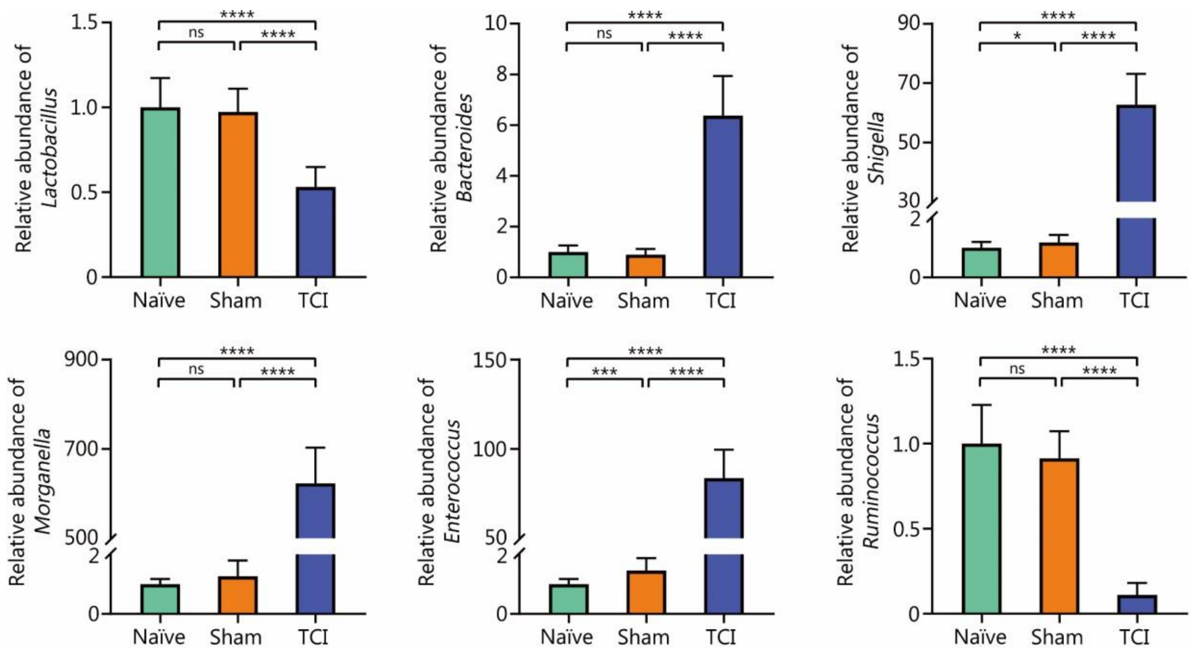

Fig. S2 Confirmation of microbiota dysregulation induced by TCI. qRT-PCR analysis to show the relative abundances of bacterial genera in the intestinal contents of rats in the naïve (n = 15), sham (n = 15) and TCI (n = 13) groups. $^{*}P < 0.05$, $^{***}P < 0.001$, $^{****}P < 0.0001$, ns non-significant. TCI traumatic colon injury, qRT-PCR quantitative real-time PCR

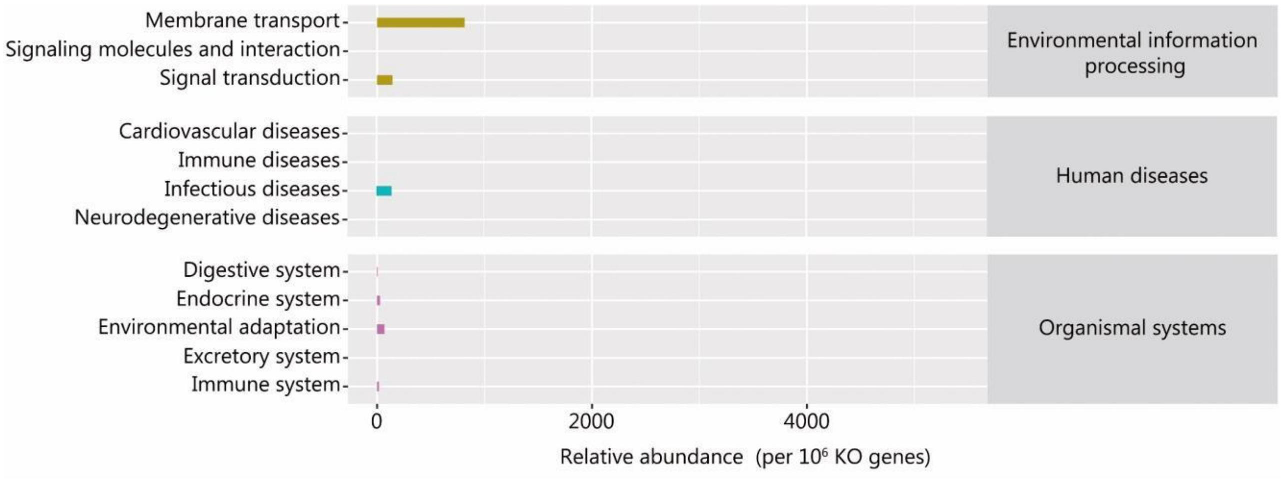

Fig. S3 KEGG analysis to show the associated pathways in the environmental information processing, human diseases and organismal systems categories based on 16S rRNA sequencing data. KEGG Kyoto Encyclopedia of Genes and Genomes, KO KEGG orthology

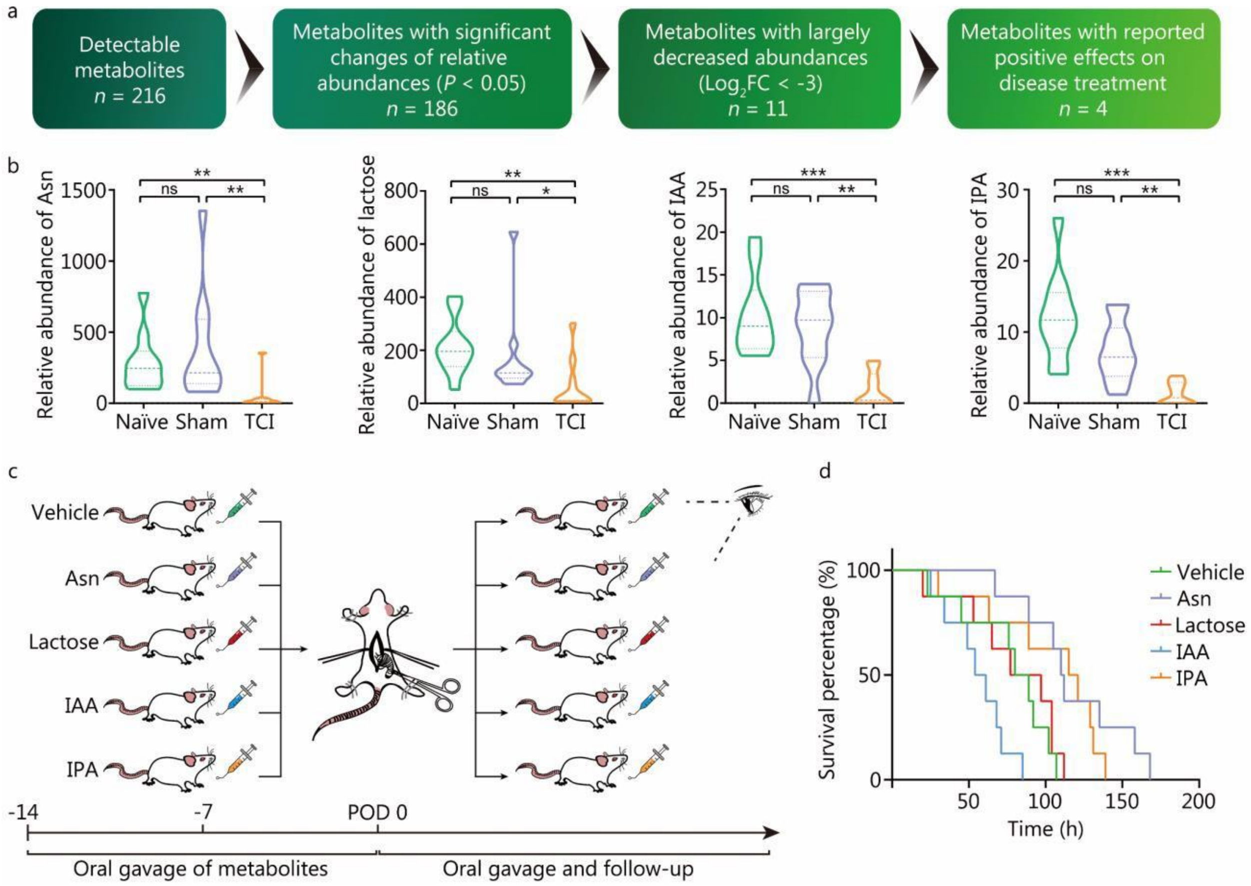

Fig. S4 Oral gavage of Asn or IPA extends the survival time of TCI rats. **a** The flow diagram to display the screening criteria and results of metabolites used for subsequent experiments. **b** The relative abundances of Asn, lactose, IAA and IPA based on the metabolomics profiling results. **c** The flow diagram to depict the experimental processes of preparing TCI rats that were administered with vehicle (n = 8), Asn (n = 8), lactose (n = 8), IAA (n = 8) or IPA (n = 8). **d** The survival curve to show the survival time of the rats as in (**c**). $^{*}P < 0.05$, $^{**}P < 0.01$, $^{***}P < 0.001$, ns non-significant. Asn asparagine, IPA 3-indolepropionic acid, IAA indoleacetic acid, FC fold change, TCI traumatic colon injury, POD post-operative day

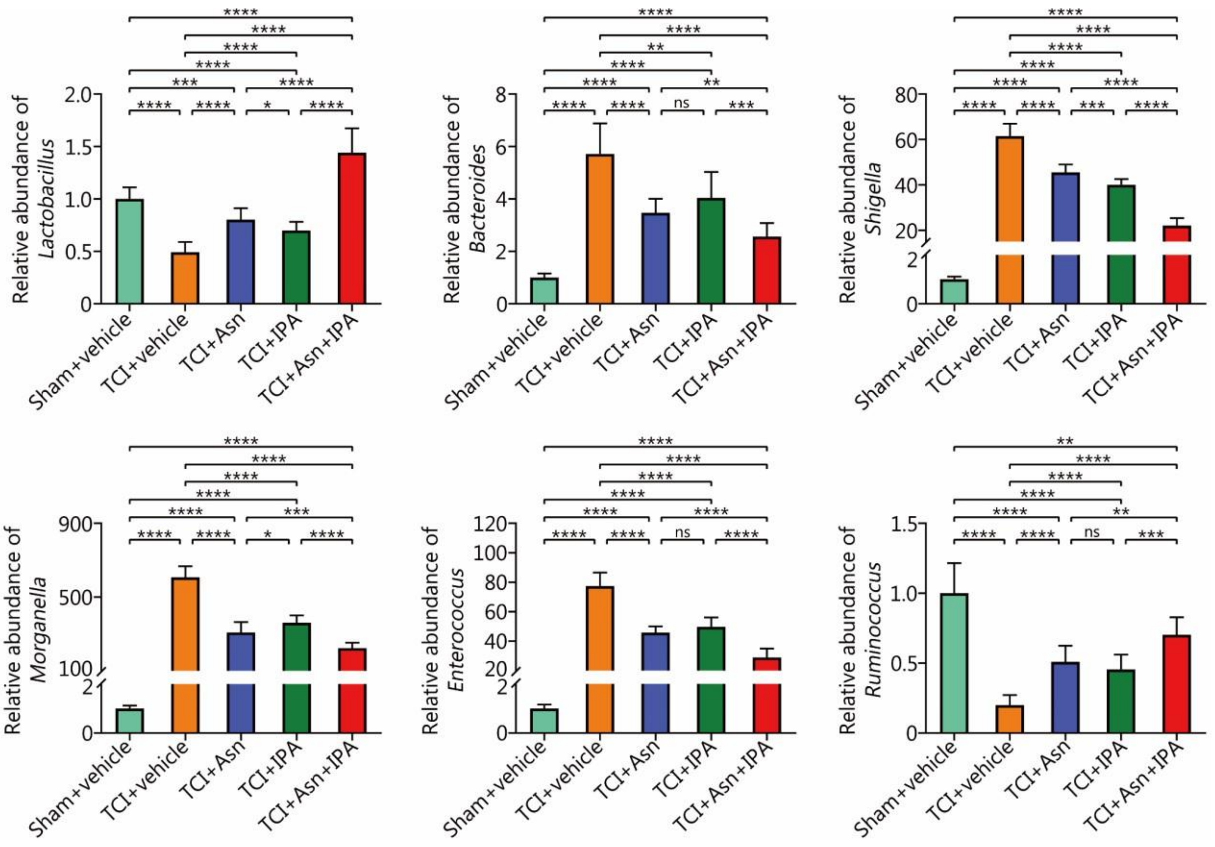

Fig. S5 Administration of Asn plus IPA mitigates intestinal microbiota dysbiosis induced by TCI. qRT-PCR analysis to show the relative abundances of bacterial genera in the intestinal contents of rats with sham operation that were administered with vehicle (n = 10) and TCI rats that were administered with vehicle (n = 9), Asn (n = 10), IPA (n = 10) or Asn plus IPA (n = 10). $^{*}P < 0.05$, $^{**}P < 0.01$, $^{***}P < 0.001$, $^{****}P < 0.0001$, ns non-significant. TCI traumatic colon injury, Asn asparagine, IPA 3-indolepropionic acid, qRT-PCR quantitative real-time PCR

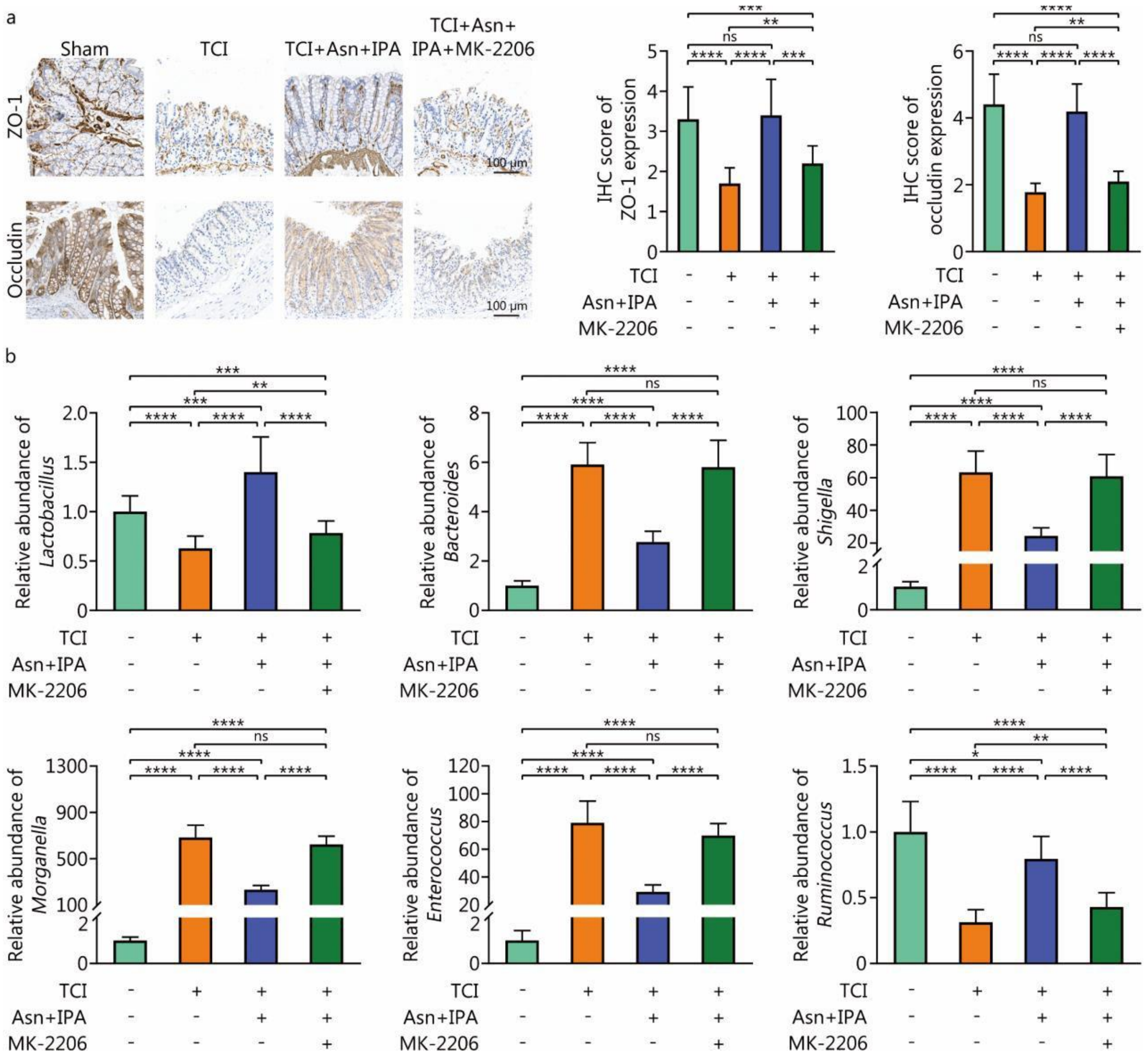

Fig. S6 Asn and IPA supplementation ameliorates intestinal hyperpermeability and intestinal microbiota dysbiosis induced by TCI through Akt activation in intestinal epithelium. **a** IHC analysis to detect ZO-1 and occludin expression in intestinal tissues of rats with sham operation that were administered with vehicle (n = 15) and TCI rats that were administered with vehicle (n = 12), Asn plus IPA (n = 15) or Asn plus IPA and MK-2206 (n = 13). The histograms of IHC scores are on the right of IHC pictures. **b** qRT-PCR analysis to show the relative abundances of bacterial genera in the intestinal contents of rats as in (**a**). $^{*}P < 0.05$, $^{**}P < 0.01$, $^{***}P < 0.001$, $^{****}P < 0.0001$, ns non-significant. TCI traumatic colon injury, Asn asparagine, IPA 3-indolepropionic acid, ZO-1 zonula occludens 1, IHC immunohistochemical, qRT-PCR quantitative real-time PCR

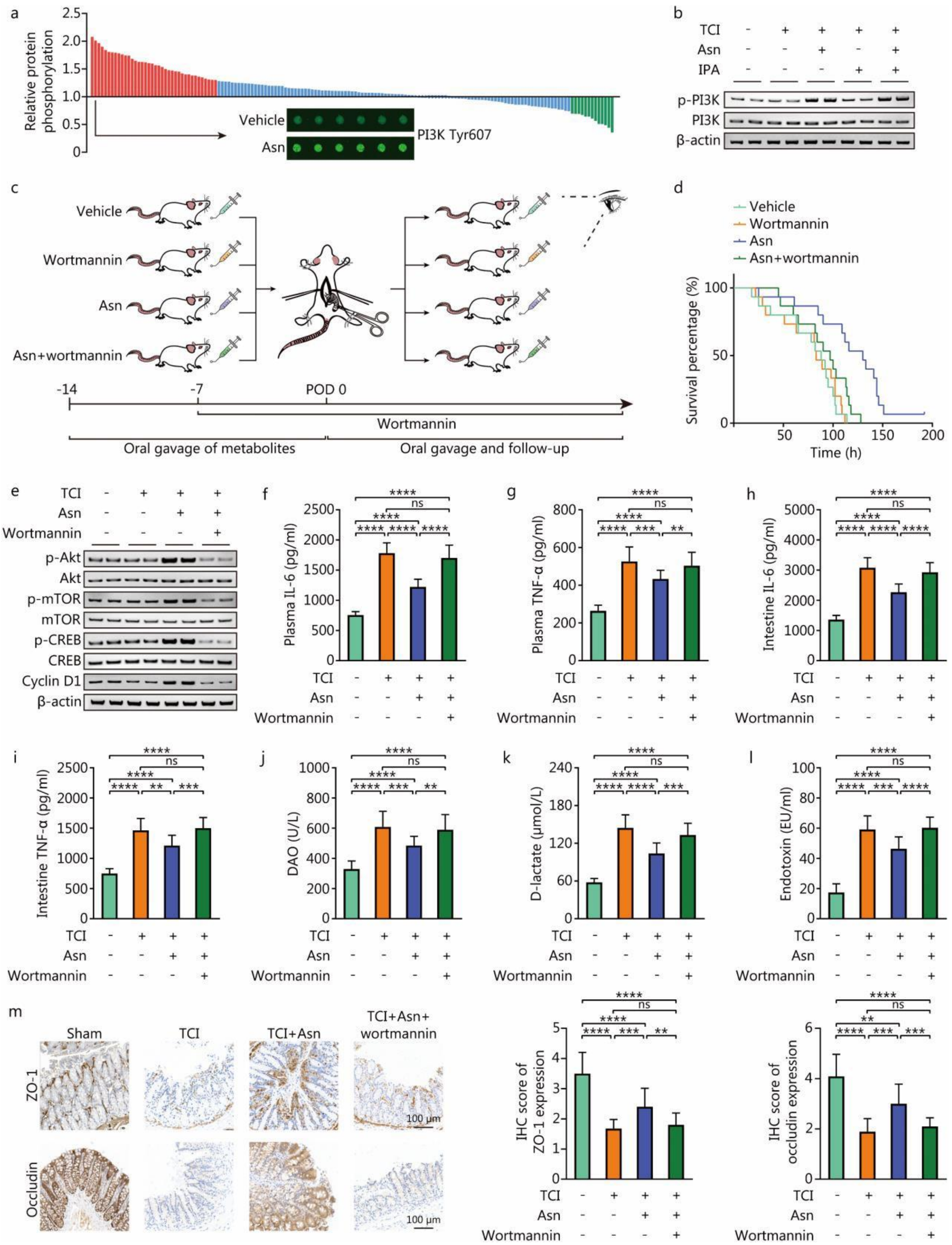

Fig. S7 Oral administration of Asn prolongs survival time and promotes intestinal recovery through activating PI3K/Akt pathway in intestinal epithelium. **a** The histogram to show protein phosphorylation with increased (FC ⩾ 1.3, red), decreased (FC ⩽ 0.7, green) and unchanged levels (0.7 < FC < 1.3, blue). PI3K Tyr607 phosphorylation

levels are compared between vehicle and Asn groups. **b** Western blotting analysis to show protein expression and phosphorylation of intestinal epithelium of rats that received indicated treatment. **c** The flow diagram to depict the experimental processes of preparing TCI rats that were administrated with vehicle, wortmannin, Asn or Asn plus wortmannin (*n* = 15 per group). **d** The survival curve to show the survival time of the rats as in (**c**). **e** Western blotting analysis to show protein expression and phosphorylation of intestinal epithelium of rats with sham operation that were administrated with vehicle (*n* = 15) and TCI rats that were administrated with vehicle (*n* = 13), Asn (*n* = 15) or Asn plus wortmannin (*n* = 13). **f-l** ELISA to detect indicator concentrations of rats as in (**e**). **m** IHC analysis to detect ZO-1 and occludin expression in intestinal tissues of rats as in (**e**). The histograms of IHC scores are on the right of IHC pictures. $^{**}P < 0.01$, $^{***}P < 0.001$, $^{****}P < 0.0001$, ns non-significant. TCI traumatic colon injury, Asn asparagine, IPA 3-indolepropionic acid, IL-6 interleukin-6, TNF-α tumor necrosis factor-α, DAO diamine oxidase, PI3K phosphoinositide 3-kinase, mTOR mammalian target of rapamycin, CREB cAMP-response element binding protein, ZO-1 zonula occludens 1, ELISA enzyme-linked immunosorbent assay, IHC immunohistochemical, POD post-operative day, FC fold change

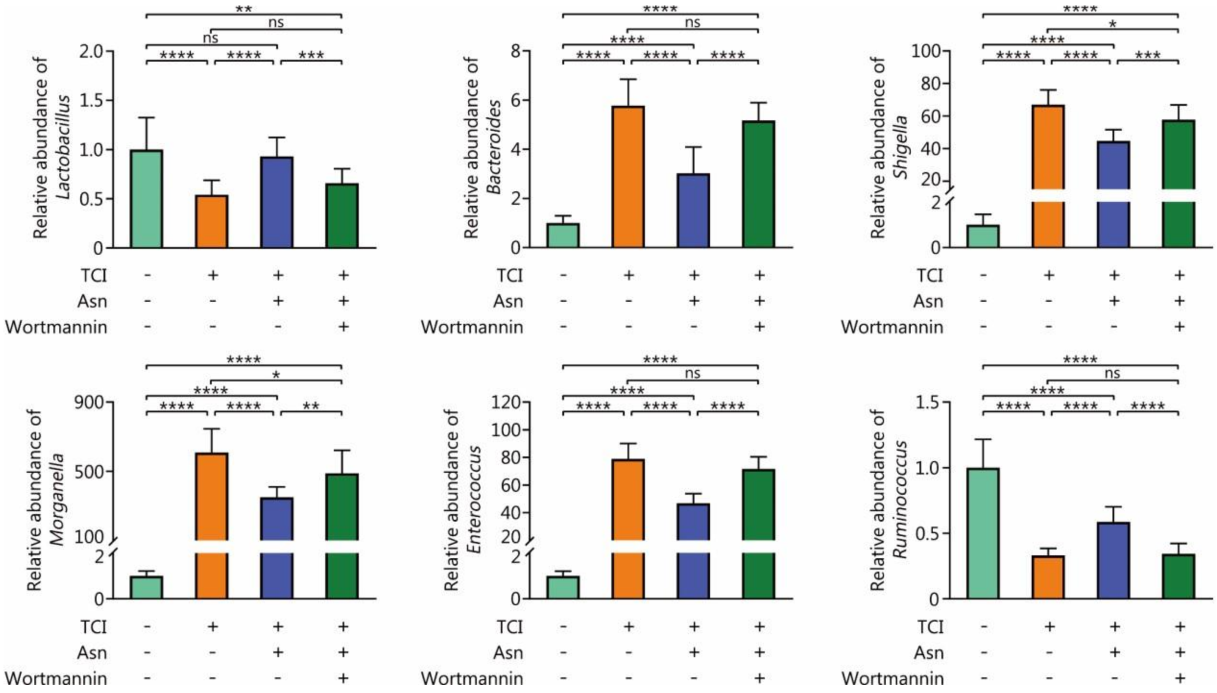

Fig. S8 Asn administration mitigates intestinal microbiota dysbiosis through phosphorylating PI3K in intestinal epithelium. qRT-PCR analysis to show the relative abundances of bacterial genera in the intestinal contents of rats with sham operation that were administrated with vehicle ($n = 15$) and TCI rats that were administrated with vehicle ($n = 13$), Asn ($n = 15$) or Asn plus wortmannin ($n = 13$). $^{*}P < 0.05$, $^{**}P < 0.01$, $^{***}P < 0.001$, $^{****}P < 0.0001$, ns non-significant. TCI traumatic colon injury, Asn asparagine, PI3K phosphoinositide 3-kinase, qRT-PCR quantitative real-time PCR

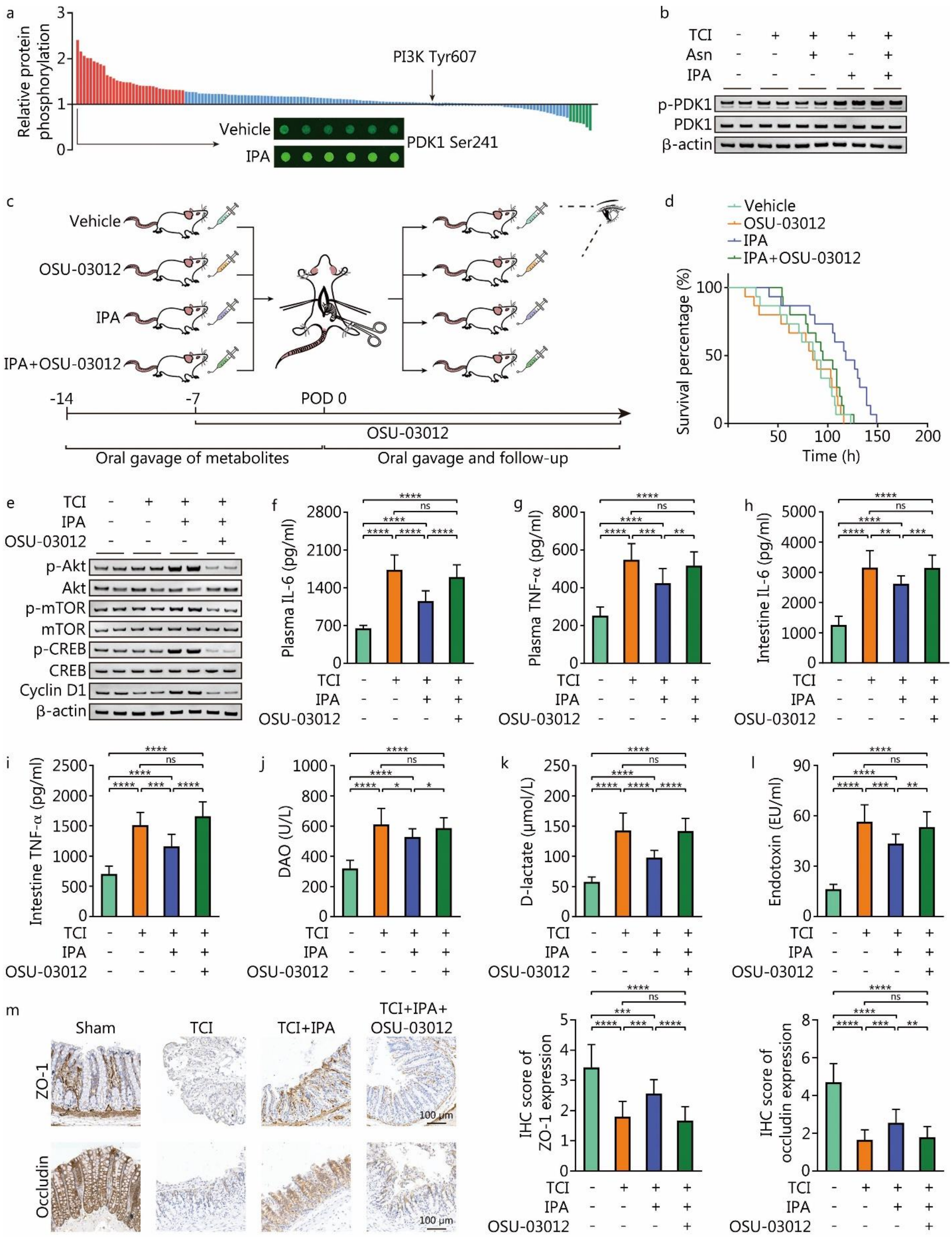

Fig. S9 Oral administration of IPA prolongs survival time and restores intestinal functions through activating PDK1/Akt pathway in intestinal epithelium. **a** The histogram to show proteins with increased (FC ≥ 1.3, red), decreased (FC ≤ 0.7, green) and unchanged phosphorylation levels (0.7 < FC < 1.3, blue). PDK1 Ser241

phosphorylation levels are compared between vehicle and IPA groups. **b** Western blotting analysis to show protein expression and phosphorylation of intestinal epithelium of rats that received indicated treatment. **c** The flow diagram to depict the experimental processes of preparing TCI rats that were administrated with vehicle, OSU-03012, IPA or IPA plus OSU-03012 ($n = 15$ per group). **d** The survival curve to show the survival time of the rats as in (**c**). **e** Western blotting analysis to show protein expression and phosphorylation of intestinal epithelium of rats with sham operation that were administrated with vehicle ($n = 15$) and TCI rats that were administrated with vehicle ($n = 14$), IPA ($n = 14$) or IPA plus OSU-03012 ($n = 14$). **f-l** The ELISA to detect indicator concentrations of rats as in (**e**). **m** IHC analysis to detect ZO-1 and occludin expression in intestinal tissues of rats as in (**e**). The histograms of IHC scores are on the right of IHC pictures. $^{*}P < 0.05$, $^{**}P < 0.01$, $^{***}P < 0.001$, $^{****}P < 0.0001$, ns non-significant. TCI traumatic colon injury, Asn asparagine, IPA 3-indolepropionic acid, IL-6 interleukin-6, TNF-α tumor necrosis factor-α, DAO diamine oxidase, PI3K phosphoinositide 3-kinase, PDK1 3-phosphoinositide dependent kinase 1, mTOR mammalian target of rapamycin, CREB cAMP-response element binding protein, ZO-1 zonula occludens 1, ELISA enzyme-linked immunosorbent assay, IHC immunohistochemical, POD post-operative day, FC fold change

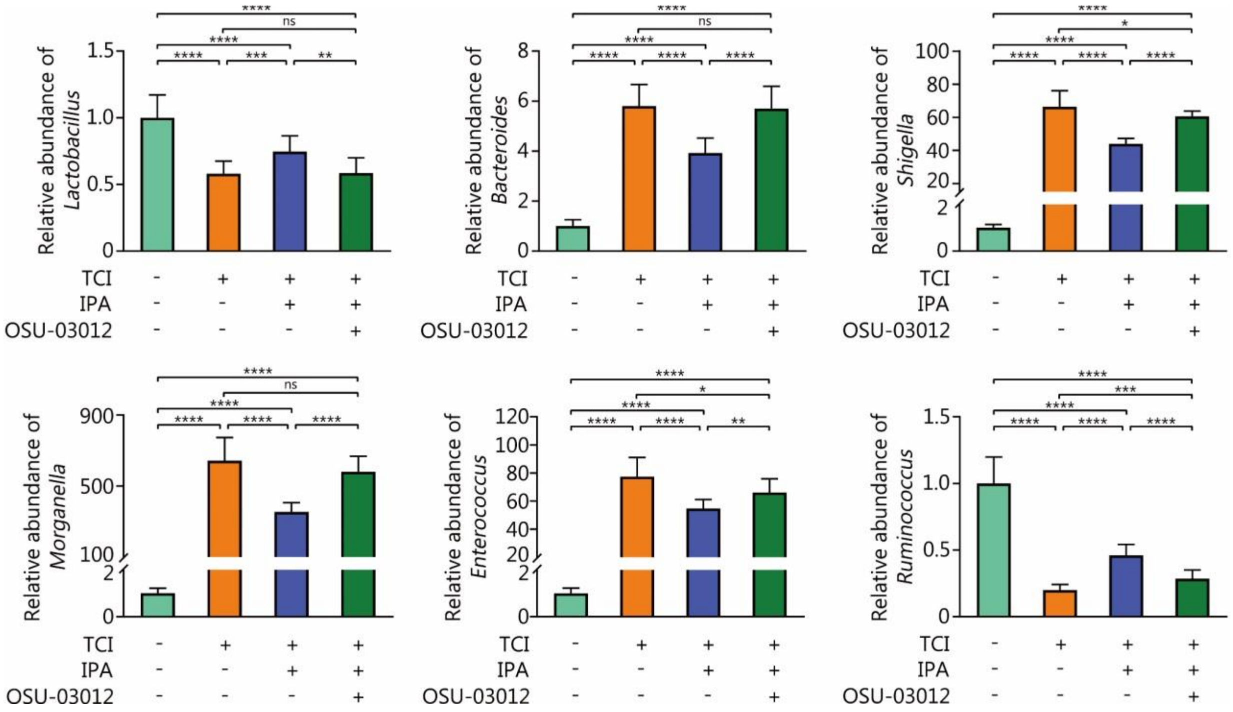

Fig. S10 IPA administration mitigates intestinal microbiota dysbiosis through phosphorylating PDK1 in intestinal epithelium. qRT-PCR analysis to show the relative abundances of bacterial genera in the intestinal contents of rats with sham operation that were administrated with vehicle (n = 15) and TCI rats that were administrated with vehicle (n = 14), IPA (n = 14) or IPA plus OSU-03012 (n = 14). $^{*}P < 0.05$, $^{**}P < 0.01$, $^{***}P < 0.001$, $^{****}P < 0.0001$, ns non-significant. TCI traumatic colon injury, IPA 3-indolepropionic acid, PDK1 3-phosphoinositide dependent kinase 1, qRT-PCR quantitative real-time PCR
